# Dimethyl fumarate and mitochondrial physiology: implications for neurological disorders

**DOI:** 10.3389/fphar.2026.1748360

**Published:** 2026-02-12

**Authors:** Marcos Roberto de Oliveira

**Affiliations:** 1 Grupo de Estudos em Neuroquímica e Neurobiologia de Moléculas Bioativas, Departamento de Química, Universidade Federal de Mato Grosso (UFMT), Cuiaba, Mato Grosso, Brazil; 2 Grupo de Estudos em Terapia Mitocondrial, Instituto de Ciências Básicas da Saúde (ICBS), Universidade Federal do Rio Grande do Sul (UFRGS), Porto Alegre, Rio Grande do Sul, Brazil; 3 Programa de Pós-Graduação em Alimentação, Nutrição e Saúde (PPGANS), Faculdade de Medicina, Universidade Federal do Rio Grande do Sul (UFRGS), Porto Alegre, Rio Grande do Sul, Brazil; 4 Programa de Pós-Graduação em Ciências Biológicas, Bioquímica, Universidade Federal do Rio Grande do Sul (UFRGS), Porto Alegre, Rio Grande do Sul, Brazil

**Keywords:** dimethyl fumarate, mitochondria, mitochondrial biogenesis, mitochondrial function, mitophagy

## Abstract

Dimethyl fumarate (DMF; C_6_H_8_O_4_) is an ester of fumaric acid widely used in clinical practice for the treatment of relapsing forms of multiple sclerosis and plaque psoriasis. Beyond its established immunomodulatory actions, DMF is increasingly recognized as a small molecule capable of reshaping cellular redox homeostasis and mitochondrial physiology. Mitochondria are double-membrane organelles that integrate energy metabolism, calcium buffering, and apoptosis regulation, while also generating reactive oxygen species that function as signaling mediators. Given their central role in neuronal survival and function, mitochondrial integrity is a critical determinant of neuroprotection. The aim of this review is to discuss the mechanistic aspects by which DMF influences mitochondrial physiology in central nervous system (CNS) cells, based on evidence from experimental models and patient-derived samples. Data consistently show that DMF activates the Nrf2 pathway, leading to increased expression of antioxidant enzymes (*e.g.*, NQO-1, HO-1) and induction of mitochondrial biogenesis markers (*e.g.*, PGC-1α, NRF1, TFAM). In neurons and oligodendrocytes, DMF enhances respiratory function and limits apoptosis by modulating BCL-2 family proteins and suppressing cytochrome c release. Disease-relevant studies further demonstrate frataxin upregulation in Friedreich’s ataxia and reduction of mitochondrial reactive oxygen species in C9orf72-related models. Conversely, in microglia, T cells, and vascular cells, DMF may impair mitochondrial respiration or increase apoptosis, particularly under inflammatory stress, suggesting a context-dependent effect. In conclusion, DMF exerts multifaceted and cell type–specific actions on mitochondria. Understanding these mechanisms may guide optimized therapeutic strategies and the identification of biomarkers for precision use in neurological disorders.

## Graphical Abstract

**Figure:**
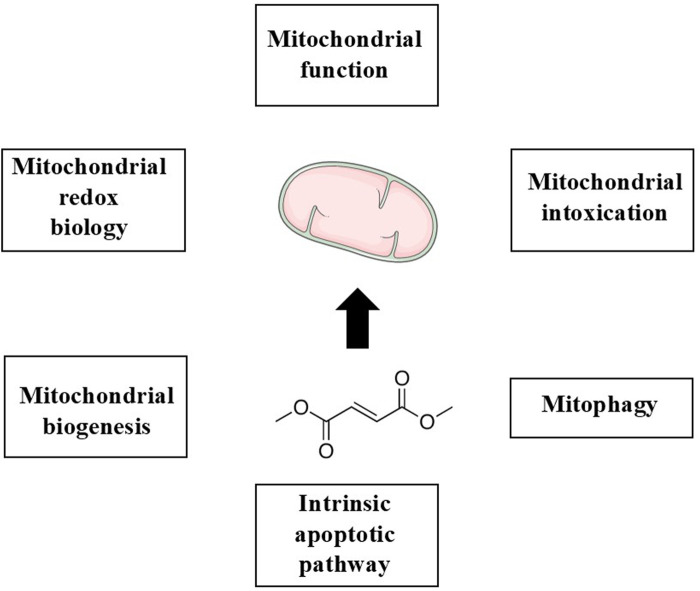
DMF modulates various biological phenomena related to mitochondrial physiology. This drug can increase or decrease mitochondrial activity, both effects being of pharmacological interest. Furthermore, DMF alters mitochondrial redox biology, potentially promoting or attenuating the production of reactive radical and non-radical species in these organelles. DMF also induces mitochondrial biogenesis (*i.e.*, synthesis of new mitochondria) and mitophagy (*i.e.*, degradation of mitochondria), depending on the context. However, it is known that DMF can act as a mitochondrial toxicant, promoting deleterious effects in healthy cells. Cell fate is modulated by DMF through a mitochondria-dependent action. Although much is known about the mechanisms of action by which DMF interferes with mitochondrial physiology, much remains to be discovered, which may allow this drug to be used in other clinical conditions in which mitochondria play a pathophysiological role. This figure was created by utilizing an image obtained from Servier Medical Art, licensed under a Creative Commons Attribution 4.0 Unported License (https://creativecommons.org/licenses/by/4.0/).

## 1 Introduction

Dimethyl fumarate (DMF) is an α,β-unsaturated ester of fumaric acid, with the molecular formula C_6_H_8_O_4_ and a molecular weight of 144.13 g/mol. Its electrophilic double bond makes it particularly reactive toward nucleophiles, allowing covalent modification of cysteine residues in proteins and glutathione through Michael addition reactions (Majkutewicz, 2022; Bresciani et al., 2023). Despite its pharmacological importance, DMF itself is poorly soluble in water and undergoes rapid hydrolysis in the small intestine, generating its active metabolite, monomethyl fumarate (MMF). Unlike DMF, MMF is detectable in plasma, reaching peak levels within 2–4 h of ingestion. Co-administration with food delays, but does not diminish, its absorption (Majkutewicz, 2022; Blair, 2018). MMF is partly protein-bound and is metabolized to fumaric acid, which enters the tricarboxylic acid cycle before elimination, mainly *via* exhaled CO_2_ (Bresciani et al., 2023; Blair, 2018). This chemical and pharmacokinetic profile underlies the suitability of DMF as an orally available therapeutic, paving the way for its diverse clinical applications.

Initially introduced in Germany in the 1950s for the treatment of psoriasis, DMF remains a first-line systemic therapy for moderate to severe plaque psoriasis (Blair, 2018). Its therapeutic spectrum expanded substantially with the 2013 approval of DMF as a first-line oral agent for relapsing-remitting multiple sclerosis (RRMS), where it demonstrated significant reductions in relapse frequency, magnetic resonance imaging (MRI) lesions, and disability progression (Fox et al., 2014; Burness and Deeks, 2014). Importantly, interest in DMF has recently shifted beyond these indications. Preclinical and clinical investigations suggest benefits in neurodegenerative diseases such as Alzheimer’s, Parkinson’s, Huntington’s, and amyotrophic lateral sclerosis (ALS), as well as in systemic sclerosis, gut disorders, and cancer models (Majkutewicz, 2022; Bresciani et al., 2023; Manai et al., 2023). The breadth of these applications highlights the pleiotropic actions of DMF and its potential as a drug suitable for repurposing across disease contexts.

The core mechanism by which DMF exerts its effects is through activation of the nuclear factor erythroid 2-related factor 2 (Nrf2) pathway. By modifying cysteine residues in Kelch-like ECH-associated protein 1 (Keap1), DMF liberates Nrf2, enabling nuclear translocation and transcription of antioxidant and cytoprotective genes such as heme oxygenase-1 (HO-1) and NAD(P)H quinone oxidoreductase-1 (NQO1) (Bresciani et al., 2023; Fox et al., 2014; Burness and Deeks, 2014). These actions strengthen glutathione synthesis and enhance resistance to oxidative stress. In parallel, DMF suppresses the nuclear factor-κB (NF-κB) pathway, reducing the production of pro-inflammatory cytokines including interleukin-1β (IL-1β), tumor necrosis factor-α (TNF-α), and interleukin-6 (IL-6) (Majkutewicz, 2022; Burness and Deeks, 2014; Gillard et al., 2015). This dual antioxidant and anti-inflammatory profile is complemented by additional mechanisms: MMF serves as an agonist of the hydroxycarboxylic acid receptor 2 (HCAR2), modulating immune metabolism *via* the adenosine monophosphate (AMP)-activated protein kinase/sirtuin 1 (AMPK/SIRT1) axis (Majkutewicz, 2022; Chen et al., 2014). DMF also succinates glycolytic enzymes such as glyceraldehyde-3-phosphate dehydrogenase (GAPDH), thereby shifting immune cell metabolism away from aerobic glycolysis (Majkutewicz, 2022; Kornberg et al., 2018). Furthermore, accumulating evidence links DMF to the regulation of autophagy and mitophagy, underscoring its potential to directly influence mitochondrial quality control (Bresciani et al., 2023). Taken together, these mechanisms illustrate how DMF operates at the crossroads of redox regulation, inflammatory control, and metabolic adaptation.

Pharmacokinetic studies confirm that systemic exposure to DMF itself is negligible due to rapid hydrolysis, while MMF displays dose-proportional kinetics and a half-life of approximately 2 h (Blair, 2018; Lategan et al., 2021). Plasma concentrations peak within 2–2.5 h, and administration with food can reduce gastrointestinal discomfort without affecting systemic exposure (Bresciani et al., 2023). Importantly, the metabolism of DMF and MMF is independent of cytochrome P450 enzymes, minimizing risks of pharmacokinetic drug-drug interactions (Blair, 2018; Xu et al., 2023). These features make DMF a predictable and relatively safe oral drug, with favorable absorption and elimination profiles supporting its long-term clinical use.

The pharmacological pleiotropy of DMF is expressed through antioxidant, anti-inflammatory, and neuroprotective effects. By engaging the Nrf2 pathway, DMF enhances cellular antioxidant defenses and glutathione biosynthesis. At the same time, inhibition of NF-κB rebalances cytokine signaling, promoting a shift from pro-inflammatory Th1/Th17 responses toward anti-inflammatory Th2 phenotypes (Blair, 2018; Wu et al., 2017; Mansilla et al., 2019). Beyond immune modulation, DMF has clear implications for mitochondrial health. *In vitro* studies show that DMF and MMF preserve mitochondrial function by stabilizing mitochondrial membrane potential (MMP) and adenosine triphosphate (ATP) production while reducing oxidative injury in astrocytes and neurons (Burness and Deeks, 2014; Rosito et al., 2020). Such actions underscore the relevance of DMF as a modulator of mitochondrial physiology, particularly in disorders where redox imbalance and organelle dysfunction are central to pathogenesis.

Despite its efficacy, DMF therapy is not without challenges. The most common adverse effects are flushing and gastrointestinal symptoms, including diarrhea, abdominal pain, and nausea, especially during early treatment (Blair, 2018; Liang et al., 2020). More concerning is lymphopenia, a reduction in circulating lymphocytes that increases susceptibility to opportunistic infections such as progressive multifocal leukoencephalopathy (PML) (Majkutewicz, 2022). Other events, including headache, fatigue, and reversible liver enzyme elevations, are generally manageable with careful monitoring (Liang et al., 2020). Thus, while DMF is generally well tolerated, long-term safety requires vigilance through laboratory and clinical follow-up.

Although DMF is well recognized as an immunomodulatory and antioxidant therapy, its broader role in mitochondrial physiology remains less defined. The purpose of this review is to critically examine how DMF influences mitochondrial physiology, including redox biology, bioenergetics, cell fate decisions, mitochondrial biogenesis, dynamics (fusion and fission), and mitophagy. By integrating evidence across these processes, this review aims to clarify how DMF-mediated signaling converges on mitochondrial homeostasis, offering new insights into its translational potential for disorders driven by mitochondrial dysfunction. DMF-induced mitochondrial intoxication is also discussed in this work.

## 2 Overview of mitochondrial physiology

**FIGURE 1:**
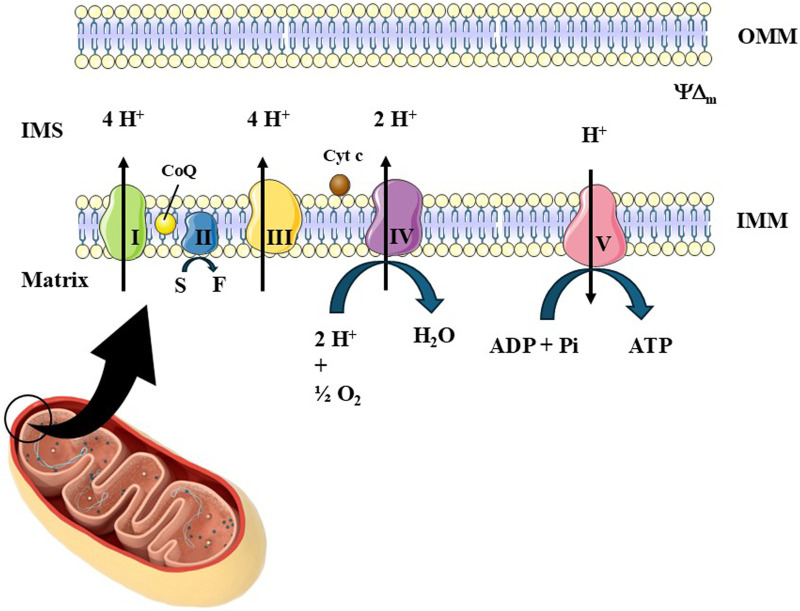
The oxidative phosphorylation system (OXPHOS). The most important role of OXPHOS is to generate ATP. This system is formed by the electron transport chain (ETC.) and Complex V (also known as ATP synthase or ATPase). The ETC, in turn, is composed of Complexes I (NADH dehydrogenase), II (succinate dehydrogenase), III (coenzyme Q:cytochrome c-oxidoreductase or cytochrome bc_1_) and IV (cytochrome c oxidase). These complexes are responsible for maintaining, together with the action of mobile elements [such as coenzyme Q_10_/ubiquinone (CoQ) and cytochrome c (Cyt c)], the flow of electrons from various sources to oxygen gas, which, when reduced by Complex IV, generates water. Complex I receives electrons from the reduced form of nicotinamide adenine dinucleotide (NADH). Complex II receives electrons from succinate (S), which is derived from the Krebs cycle (also called the tricarboxylic acid cycle), generating fumarate (F) and reducing flavin adenine dinucleotide (FAD) into FADH_2_. CoQ receives electrons from the Complexes I and II and can also be reduced by electrons originated from the oxidation of fatty acids and glucose (through the work of the electron shuttles). Cyt c carries one electron at a time from Complex III to Complex IV. As electrons flow through the ETC, Complexes I, III, and IV pump protons (H^+^) into the intermembrane space (IMS), generating an electrochemical gradient across the IMM. Complex V uses the movement of H^+^ from the IMS to the mitochondrial matrix to generate ATP from ADP and inorganic phosphate (Pi).

The double-membrane organelles mitochondria are the major source of adenosine triphosphate (ATP) and take a role in several other metabolic pathways and biological phenomena necessary to the maintenance of the viability of animal and vegetal cells (Harrington et al., 2023). The oxidative phosphorylation (OXPHOS) system, which is responsible for the ATP production, is found in the inner mitochondrial membrane (IMM) and is composed of the electron transport chain (ETC.) components (Complex I–IV and the mobile elements coenzyme Q10/ubiquinone and cytochrome c) and of Complex V (ATP synthase/ATPase) (Figure 1) (Papa et al., 2012; Rasheed and Tarjan, 2018; Zorova et al., 2018; Kondadi et al., 2020; Tang et al., 2020; Joubert and Puff, 2021; Harrington et al., 2023). Mitochondria can undergo structural and functional changes through events such as mitochondrial biogenesis, mitochondrial fusion and fission, and mitophagy, as described below.

### 2.1 Mitochondrial biogenesis

**FIGURE 2:**
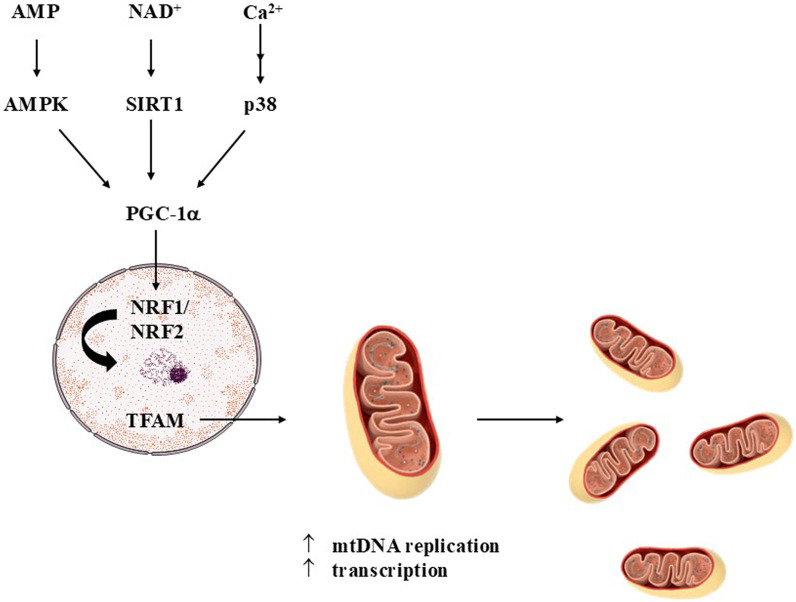
Mitochondrial biogenesis. The synthesis of new mitochondria is stimulated by several factors, including adenosine monophosphate (AMP), nicotinamide adenine nucleotide (NAD^+^, oxidized form), and calcium ions (Ca^2+^), among others. These signaling agents activate AMP-activated protein kinase (AMPK), sirtuin 1 (SIRT1), and calcium/calmodulin-dependent protein kinase (CaMK, that stimulates p38). In the next steps of each signaling pathway, the peroxisome proliferator-activated receptor-gamma coactivator-1α (PGC-1α), a transcription coactivator, is activated and translocates to the nucleus of the cell. Then, the transcription of nuclear respiratory factors 1 and 2 (NRF1 and NRF2, respectively) is promoted, leading an upregulation in the expression of mitochondrial transcription factor A (TFAM). TFAM migrates to the mitochondria, in which is enhances the synthesis of mitochondrial DNA (mtDNA) and the transcription of proteins associated with the oxidative phosphorylation (OXPHOS) system. The next steps conclude the synthesis of new mitochondria in the cells. Figure created by using an image obtained from Servier Medical Art, licensed under a Creative Commons Attribution 4.0 Unported License (https://creativecommons.org/licenses/by/4.0/).

Mitochondrial biogenesis (also called mitobiogenesis or mitogenesis), i.e., the synthesis of new mitochondria, can be stimulated by different endogenous agents, including adenosine monophosphate (AMP), oxidized nicotinamide adenine dinucleotide (NAD^+^), cyclic AMP (cAMP), and calcium ions (Ca^2+^), among other signals (Pfanner et al., 2019) (Figure 2). In that regard, mitochondrial biogenesis is part of the cellular adaptation to exercise, caloric restriction, temperature decline, and diseases, among other physiological contexts (Jornayvaz and Shulman, 2010; Wang et al., 2023). Increased AMP amounts, which can indicate low ATP levels due to decreased production and/or increased consumption, promote the activation of AMP-activated protein kinase (AMPK), that phosphorylates the peroxisome proliferator-activated receptor-gamma coactivator-1α (PGC-1α), a transcription coactivator that translocates to the cell nucleus and stimulates the expression of the nuclear respiratory factors 1 and 2 (NRF1 and NRF2, respectively) (Jäger et al., 2007; Fernandez-Marcos and Auwerx, 2011). In the nucleus, NRF1 and NRF2 promote the expression of mitochondrial transcription factor A (TFAM), a protein that induces the transcription of specific targets in the mitochondria, as well as promotes mitochondrial DNA (mtDNA) replication and condensation (Farge et al., 2014; Kukat et al., 2015). PGC-1α can be activated also by SIRT1, a deacetylase, which is upregulated by NAD^+^ (Gerhart-Hines et al., 2007; Rodgers et al., 2005). AMPK also upregulates SIRT1 by increasing the levels of NAD^+^ (Cantó et al., 2009; Fulco et al., 2008). On the other hand, Ca^2+^ modulate PGC-1α by a mechanism dependent on Ca^2+^/calmodulin-dependent protein kinase (CaMK) and p38 protein kinase, also leading to mitochondrial biogenesis (Wright et al., 2007). The modulation of mitochondrial biogenesis by cAMP initiates with the activation of protein kinase A (PKA), followed by the phosphorylation of cAMP response element-binding protein (CREB), that leads to the expression of PGC-1α (Bouchez and Devin, 2019). Mitochondrial biogenesis alters the number, size and mass of the organelles, allowing bioenergetic improvements to respond to stressful conditions (Jornayvaz and Shulman, 2010).

### 2.2 Mitochondrial fusion and fission

**FIGURE 3:**
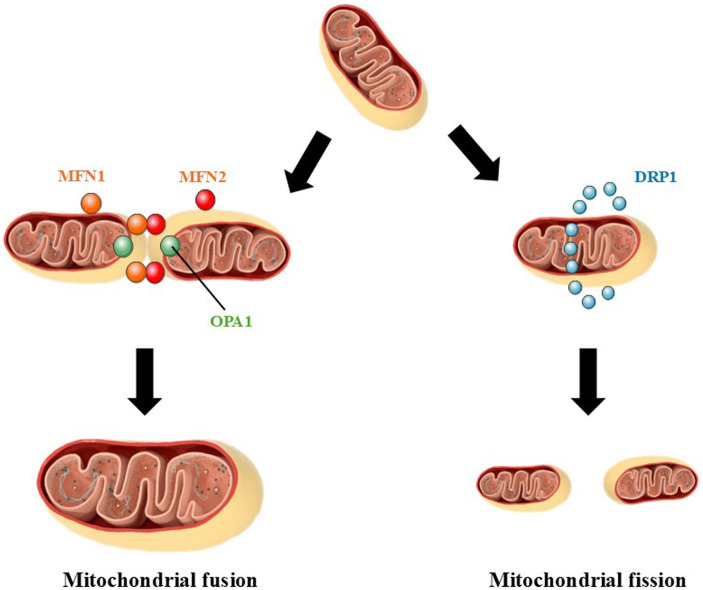
Mitochondrial fusion and fission. In mitochondrial fusion, two mitochondria fuse generating one larger mitochondrion. This phenomenon is dependent on the proteins mitofusin 1 (MFN1) and mitofusin 2 (MFN2) located in the outer mitochondrial membrane (OMM). MFN proteins mediate the binding and docking of two mitochondria. The fusion of the inner mitochondrial membrane (IMM) is mediated by optic atrophy 1 protein (OPA1) by a mechanism associated with cardiolipin. Mitochondrial fission, on other hand, is the event in which one mitochondrion undergoes fission generating two smaller mitochondria. Fission of one mitochondrion is started by an interaction with the endoplasmic reticulum, which causes begins to constrict the mitochondrion. This step is followed by the recruitment of dynamin-related protein 1 (DRP1) by mitochondrial fission 1 protein (FIS1) to the OMM. Oligomerization of DRP1 leads to the formation of a ring-like structure around the mitochondrion. Other proteins, such as dynamin 2 (DNM2), are recruited to continue the fission of the mitochondrion.

Mitochondrial fusion (combination of two separate mitochondria leading to the formation of a large organelle) and fission (mitochondrial fragmentation leading to the formation of separate organelles) lead to alterations in the number, form, and size of mitochondria (Shen et al., 2023) (Figure 3). Mitochondrial fusion initiates after the interaction of two mitochondria and the fusion of the OMM in an event that is coordinated by mitofusins 1 and 2 (MFN1 and MFN2, respectively), which are dependent of guanosine triphosphate (GTP) (Dorn, 2020). After the fusion of the OMM, the IMM of each mitochondria initiates their combination in a process dependent on optic atrophy 1 protein (OPA1), another GTPase (Tilokani et al., 2018; Shen et al., 2023). Cardiolipin, a phospholipid present at high concentrations in the IMM, cooperates with the OPA1-dependent fusion of the IMM (Ban et al., 2018). Mitochondrial fusion is stimulated, for example, when a mitochondrion begins to present dysfunctions (*e.g.*, decreased ATP production, increased production of reactive species) or increase in the levels of markers of chemical damage (such as mtDNA damage and lipid peroxidation) (Chen et al., 2023). Thus, a healthy mitochondrion (which does not present high levels of markers of molecular damage, therefore) fuses with a dysfunctional one, distributing diverse molecular components and preventing the progression of organellar dysfunction (Chen et al., 2023).

On the other hand, mitochondrial fission leads to the separation of one mitochondrion in two mitochondria (Shen et al., 2023) (Figure 3). The endoplasmic reticulum (ER) cause the constriction of the mitochondrion by a mechanism involving nucleation and polymerization of actin at the contact sites between the organelles (de Brito and Scorrano, 2010; Friedman et al., 2011; Ji et al., 2015; Manor et al., 2015). After this step, mitochondrial fission 1 protein (FIS1) recruits dynamin-related protein 1 (DRP1), which oligomerizes and forms a ring-shaped structure around the mitochondrion (Sun et al., 2024). GTP is cleaved and dynamin 2 (DNM2) is recruited to the constriction site, leading to the fission of the OMM (Sun et al., 2024). The fission of the IMM seems to be dependent on Ca^2+^ and cardiolipin and is mediated by DRP1 in a GTP-dependent manner (Paradies et al., 2019). Two smaller mitochondria are generated after the mitochondrial fission process is completed. Stimulating mitochondrial fission is important because less functional portions of a mitochondrion are eliminated, preventing them from remaining in the organelle, which would cause, among other effects, an increase in the production of reactive species and, consequently, in the levels of redox stress markers (Chen et al., 2023). Smaller and dysfunctional mitochondria, originating from mitochondrial fission, can be degraded in the phenomenon of mitophagy (Kraus et al., 2021).

### 2.3 Mitophagy

**FIGURE 4:**
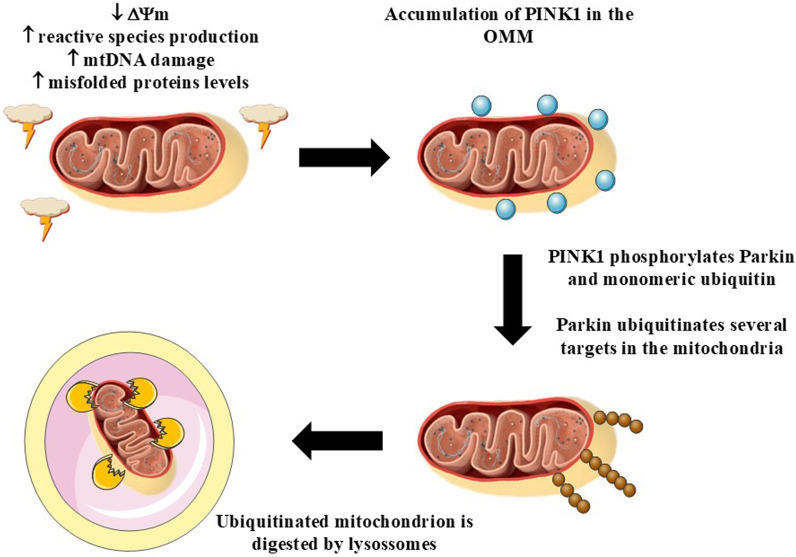
Mitophagy. Mitochondrial dysfunction, *i.e.*, loss of mitochondrial membrane potential (MMP, ΔΨ_m_), mitochondrial DNA (mtDNA) damage, increased production of reactive species, and/or enhanced levels of misfolded proteins, leads to the accumulation of (PINK1) in the outer mitochondrial membrane (OMM). PINK1 undergoes transautophosphorylation and also phosphorylates Parkin and monomeric forms of ubiquitin. The next steps include ubiquitination of several proteins located in the mitochondria, such as mitofusins 1 and 2 (MFN1 and MFN2, respectively), by Parkin. The growing ubiquitin chains in mitochondria interact with autophagy adapters, which mediate the binding of ubiquitinated mitochondria with LC3 located in the membrane of the phagophores. This interaction leads to the formation of the autophagosomes, that fuse with lysosomes that are responsible for digesting mitochondria. Figure created by using an image obtained from Servier Medical Art, licensed under a Creative Commons Attribution 4.0 Unported License (https://creativecommons.org/licenses/by/4.0/).

Mitochondrial fission may or not be followed by mitophagy, a phenomenon in which damaged mitochondria is digested by the cell (Wang et al., 2023) (Figure 4). Loss of MMP, mtDNA damage, augmented reactive species production, and accumulation of misfolded proteins, for example, trigger mitophagy by a mechanism associated with the accumulation of serine/threonine PTEN-induced putative kinase 1 (PINK1) in the OMM, followed by its transautophosphorylation at Ser228 (Gan et al., 2022). Then, PINK1 phosphorylates Parkin, an E3 ubiquitin protein ligase, and monomeric ubiquitin, which, in turn, promotes amplification of the activation of Parkin (Harper et al., 2018). Activated Parkin ubiquitinates proteins located in the OMM, including the voltage-dependent anion channel 1 (VDAC1), mitochondrial Rho-GTPase 1 (Miro1), and proteins involved in the control of mitochondrial fusion, among others (Birsa et al., 2014; Tanaka et al., 2010; Wang et al., 2023). This event leads to phosphorylation of these proteins by PINK1 and to the amplification of the recruitment of Parkin to the mitochondria, causing an increase in the number of ubiquitin chains in the organelles (Harper et al., 2018). This sequence of chemical modifications allows the interaction of the mitochondria with the autophagy adapters, including sequestosome 1 (p62/SQSTM1), nuclear dot protein 52 (NDP52/CALCOCO2), and optineurin (OPTN), to cite a few (Matsumoto et al., 2011; Thurston et al., 2009; Wong and Holzbaur, 2015). The adapters mediate the interaction of ubiquitinated mitochondria with the phagophores by a mechanism related to microtubule-associated protein 1A/1B-light chain 3 (LC3) present in the membranes of these structures (Johansen and Lamark, 2020). The formation of the autophagosomes, which are double-membrane structures, leads to the fusion of the lysosomes with ubiquitinated mitochondria and the digestion of the organelles (Wang et al., 2023). Alternatively, mitophagy can be triggered by mechanisms independent on the PINK1/Parkin pathway (Wang et al., 2023).

### 2.4 Mitochondrial redox biology

In addition to being the most important source of ATP for animal cells, mitochondria constantly produce reactive oxygen species (ROS) through ETC (Masuda et al., 2024). Among the ROS, the most prominent are superoxide radical (O_2_^−•^), hydrogen peroxide (H_2_O_2_), and hydroxyl radical (^•^OH) (Lambert and Brand, 2009; Murphy, 2009; Sies et al., 2022). Furthermore, there is data showing that mitochondria can generate reactive nitrogen species (RNS), such as nitric oxide (NO^•^) and peroxynitrite (ONOO^−^), for example, (Lacza et al., 2009; De Armas et al., 2019). Mitochondrial ROS and RNS exert physiological roles in modulating signaling pathways associated with the control of bioenergetics, stress response, hypoxia adaptation, immune response, and cell death (Sena and Chandel, 2012; Sies et al., 2022; 2024). Elevated levels of ROS and/or RNS can not only cause mitochondrial dysfunction, but also widespread damage to cellular components, including proteins, lipids, and nucleic acids (Sies et al., 2017; Cardozo et al., 2023). The antioxidant defenses present in mitochondria can be of the non-enzymatic (such as glutathione - GSH) and enzymatic types (Mailloux, 2018; Marí et al., 2020; Chen et al., 2024). The latter include the enzymes manganese-dependent superoxide dismutase (Mn-SOD), catalase (CAT), different isoforms of glutathione peroxidase (GPx), and peroxiredoxin (PRx) (Cao et al., 2007; Holley et al., 2011; Huang et al., 2019).

### 2.5 Mitochondria and cell death

**FIGURE 5:**
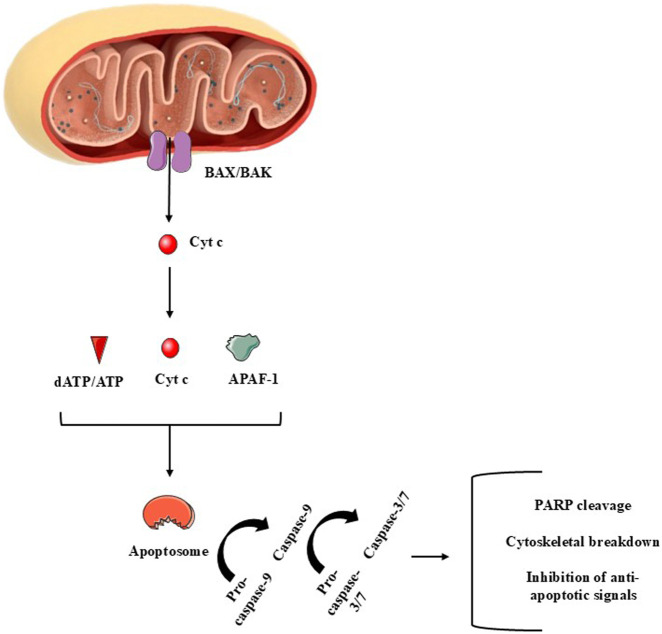
A summary of major components of the mitochondria-dependent apoptotic cell death (intrinsic apoptotic pathway). The release of cytochrome c (cyt c) to the cytoplasm is mediated by proteins such as BAX, among several others. In the cytoplasm, cyt c reacts with apoptotic peptidase activating factor-1 (APAF-1) and deoxy-ATP (dATP) or with ATP. Pro-caspase-9 is necessary to complete the apoptosome, that activates this initiator enzyme. Once activated, caspase-9 mediates the activation of caspase-3/7, which are called effector caspases due to the several apoptotic roles these enzymes perform during apoptosis. Figure created by using images obtained from Servier Medical Art, licensed under a Creative Commons Attribution 4.0 Unported License (https://creativecommons.org/licenses/by/4.0/).

Mitochondria control cell death primarily by integrating stress signals into regulated changes in mitochondrial membrane integrity, metabolism, and signaling output. The central mitochondrial mechanism governing apoptosis is mitochondrial outer membrane permeabilization (MOMP), a process tightly regulated by the B-cell lymphoma 2 (BCL-2) protein family (Bock and Tait, 2020; Czabotar and Garcia-Saez, 2023; Glover et al., 2024). In response to genotoxic, metabolic, or oxidative stress, pro-apoptotic BH3-only proteins activate the multidomain effectors BCL-2-associated X protein (BAX) and BCL2 homologous antagonist/killer (BAK), promoting their oligomerization within the outer mitochondrial membrane and formation of proteolipidic pores (Li et al., 2024; Vogler et al., 2025). MOMP enables the release of cytochrome c and other intermembrane space proteins, including second mitochondria-derived activator of caspase/direct inhibitor of apoptosis-binding protein with low pI (SMAC/DIABLO, respectively), which together activate the apoptosome [apoptotic protease activating factor 1 (APAF1)/caspase-9 complex) and downstream executioner caspases-3 and -7 (Figure 5). While caspases orchestrate the biochemical dismantling of the cell, mitochondrial dysfunction following widespread MOMP (including loss of membrane potential, impaired OXPHOS, and ATP depletion) can itself drive caspase-independent cell death, underscoring mitochondria as the point of irreversible cellular collapse (Bock and Tait, 2020; Dho et al., 2025).

Beyond canonical apoptosis, mitochondria modulate cell fate through quantitative control of MOMP. Sublethal or “minority” MOMP affects only a subset of mitochondria, generating limited caspase activity insufficient to cause immediate death (Liu et al., 2025). This partial mitochondrial permeabilization can induce DNA damage, cellular senescence, or stress-adaptive phenotypes, highlighting mitochondria as regulators of death signaling intensity rather than simple on/off switches (Hall-Younger and Tait, 2025).

Mitochondria also control inflammatory forms of cell death by releasing mitochondrial-derived damage-associated molecular patterns (DAMPs). Following extensive membrane remodeling, mitochondrial DNA, cardiolipin, and ROS can escape into the cytosol, activating innate immune pathways such as cyclic GMP-AMP synthase/stimulator of interferon genes (cGAS/STING, respectively), inflammasomes, and type I interferon signaling (Lin et al., 2022; Glover et al., 2024). Thus, mitochondrial permeabilization links cell death execution to inflammatory signaling.

Collectively, mitochondria govern cell death through coordinated regulation of membrane permeabilization, bioenergetic collapse, caspase activation, and immunogenic signaling, positioning them as central decision-making hubs in cellular fate control.

## 3 Brain vulnerability to redox stress: Implications for mitochondrial dysfunction and therapeutic modulation

The central nervous system (CNS) operates under uniquely demanding bioenergetic conditions, consuming nearly 20% of the oxygen (O_2_) originated from inspiration to sustain synaptic transmission, neurotransmitter cycling, and ion homeostasis. This high metabolic rate, coupled with limited antioxidant defenses, makes neurons particularly vulnerable to redox imbalance and mitochondrial dysfunction (Cobley et al., 2018; Șerban et al., 2025). The major sources of ROS in the CNS are mitochondria, the endoplasmic reticulum (ER), peroxisomes, and enzyme systems such as NADPH oxidases (NOX), nitric oxide synthases (NOS), xanthine oxidase (XO), and cyclooxygenases (COX) (Lee et al., 2021; Üremiş and Üremiş, 2025). Within mitochondria, the ETC, particularly Complexes I and III, is a predominant site of O_2_^−•^ generation. This O_2_^−•^ is rapidly converted by superoxide dismutases (SODs) into H_2_O_2_, which can serve as a signaling molecule or, in the presence of transition metals, form ^•^OH through Fenton chemistry (Șerban et al., 2025). NO^•^, generated by neuronal and inducible NOS (iNOS), is another critical redox mediator; however, excess NO^•^ can react with O_2_^−•^ to form ONOO^−^, a potent RNS (Üremiş and Üremiş, 2025; Ferrer-Sueta et al., 2018).

Despite constant exposure to ROS and RNS, CNS relies on an array of antioxidant defenses. Enzymatic systems include SOD isoforms [including copper/zinc-dependent superoxide dismutase (Cu/Zn-SOD) and Mn-SOD], CAT, GPx, and PRx, while non-enzymatic antioxidants include, among others, GSH, melatonin, vitamins C and E, and coenzyme Q_10_ (Șerban et al., 2025). The Nrf2 pathway orchestrates transcriptional responses to oxidative challenges by inducing antioxidant and detoxification genes (Trofin et al., 2025). However, the CNS exhibits relatively modest antioxidant capacity compared with peripheral tissues, contributing to its vulnerability (Cobley et al., 2018).

Multiple structural and metabolic features explain why the CNS is particularly sensitive to oxidative and nitrosative stress. First, neurons are post-mitotic cells with limited regenerative capacity, making accumulated oxidative damage irreversible (Șerban et al., 2025). Second, neuronal membranes are enriched in polyunsaturated fatty acids (PUFA), which are highly susceptible to lipid peroxidation, leading to toxic aldehydes such as malondialdehyde (MDA) and 4-hydroxynonenal (4-HNE) (Trofin et al., 2025). Third, the brain contains abundant redox-active metals (iron and copper), which catalyze ROS generation *via* Fenton and Haber-Weiss reactions (Cobley et al., 2018). Fourth, excitatory neurotransmitters like glutamate promote Ca^2+^ overload and excitotoxicity, further stimulating mitochondrial ROS production (Cobley et al., 2018; Trofin et al., 2025). The CNS is also impacted by both autoxidation reactions and neurotransmitter degradation, such as dopamine (Cobley et al., 2018; Umek et al., 2018). These reactions can generate both O_2_^−•^ and/or H_2_O_2_, potentially causing molecular damage and mitochondrial dysfunction, depending on the context (Cobley et al., 2018; Meiser et al., 2013). Last but not least, glucose metabolism is intense in the CNS, favoring the formation and, sometimes, accumulation of derivatives such as methylglyoxal, a highly reactive aldehyde (Cobley et al., 2018). Collectively, these factors, among others, help to explain the redox vulnerability of the CNS.

Mitochondria occupy a pivotal position in CNS redox biology, simultaneously serving as powerhouses and redox regulators (Song et al., 2024). Beyond ATP synthesis through oxidative phosphorylation, they regulate Ca^2+^ buffering, synthesize steroid hormones and neurotransmitter precursors, and govern cell fate decisions *via* apoptosis pathways (Lee et al., 2021; Song et al., 2024). Dysfunctional mitochondria not only impair bioenergetics but also amplify ROS and RNS production, creating a vicious cycle that exacerbates oxidative injury (Üremiş and Üremiş, 2025; Zorov et al., 2014). Mitochondria are the dominant, dynamically regulated source of ROS in neurons; Complexes I/III electron leak, redox-coupled shifts during Ca^2+^ uptake, and metabolic state transitions all tune H_2_O_2_ emission that can signal adaptively or tip into damage (Sies and Jones, 2020). Endoplasmic reticulum oxidative folding [which is dependent on protein disulfide isomerase (PDI) and endoplasmic reticulum oxidase 1 (ERO1)], peroxisomal oxidases, and NADPH oxidases (*e.g.*, NOX4 at multiple subcellular locales) provide additional ROS streams; inter-organelle contacts propagate redox cues (Shergalis et al., 2020). By contrast, RNS arise primarily from neuronal and glial NOS; NO^•^ modulates synaptic function but, in the presence of O_2_^−•^, forms ONOO^−^ that nitrates proteins and impairs respiration (Boczkowski et al., 1999; Brown and Borutaite, 2007). Moreover, mitochondrial-derived H_2_O_2_ and O_2_^−•^ act as signaling molecules that modulate oxidative post-translational modifications (oxPTM) of cysteine residues, influencing redox-sensitive signaling cascades relevant to neuronal survival and plasticity (Sies, 2017).

When ROS and RNS exceed the buffering capacity of antioxidant defenses, oxidative and nitrosative stress arise, damaging proteins, lipids, and DNA (Halliwell, 2006). This imbalance activates pro-inflammatory pathways, including NF-κB signaling, in glial cells, perpetuating neuroinflammation and disrupting the blood-brain barrier (BBB) (Trofin et al., 2025; Solleiro-Villavicencio and Rivas-Arancibia, 2018). Neuroinflammation, in turn, exacerbates mitochondrial dysfunction and ROS production, creating a self-sustaining loop (Lin et al., 2022; Peggion et al., 2024). Such processes are central to the pathogenesis of neurodegenerative diseases including Alzheimer’s disease, where oxidative damage contributes to amyloid-β (Aβ) aggregation and tau hyperphosphorylation; Parkinson’s disease, where oxidative modification of α-synuclein promotes Lewy body formation; multiple sclerosis, where chronic inflammation and demyelination are coupled with mitochondrial stress; and ALS, where mutations in antioxidant enzymes such as SOD1 (that codes for Cu/Zn-SOD) exacerbate ROS accumulation (Șerban et al., 2025; Üremiş and Üremiş, 2025).

Despite recognition of oxidative stress as a unifying mechanism in CNS pathology, clinical trials with broad-spectrum antioxidants have yielded disappointing outcomes (Carvalho et al., 2017; Forman and Zhang, 2021). This failure highlights the dual role of ROS and RNS as both damaging agents and essential signaling molecules (Sies, 2017). Thus, indiscriminate suppression of reactive species may disrupt physiological signaling necessary for neuronal function. A more nuanced approach, targeting specific sources of pathological ROS/RNS (*e.g.*, NOX2 in activated microglia) or restoring redox-sensitive signaling pathways (*e.g.*, Nrf2 activation by dimethyl fumarate), is likely to offer greater therapeutic promise.

## 4 The effects of DMF on mitochondrial physiology

The mechanisms of action by which DMF affects mitochondrial physiology are not completely clear yet. Moreover, some research groups have reported that DMF failed to significantly modulate mitochondria-related parameters in certain cell types (Silva et al., 2020; Szczesny-Malysiak et al., 2020). Thus, in the present work, the debate is focused on the potential therapeutic targets (*i.e.*, pharmacological candidates) whose modulation by DMF promotes benefits to mitochondria in CNS cells. Mechanistic works are of particular interest due to their importance in a clinical scenario.

### 4.1 Effects of DMF on mitochondrial function and redox biology in neurological models

**FIGURE 6:**
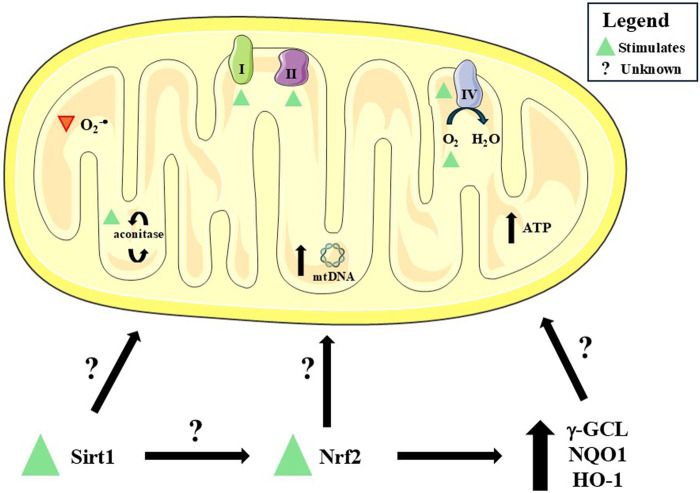
A summary of the effects induced by dimethyl fumarate (DMF) on mitochondrial function and redox biology. DMF stimulates (green triangle) aconitase (an enzyme of the tricarboxylic acid cycle) and Complexes I, II, and IV activity and the flux of oxygen (O_2_) in the mitochondria of brain cells, probably leading to enhanced adenosine triphosphate (ATP) production. DMF also caused a decrease (red triangle) in the production of radical superoxide (O_2_^−•^) by mitochondria. Additional effects include increased mitochondrial DNA (mtDNA) copy number and upregulation of sirtuin 1 (Sirt1), nuclear factor erythroid 2-related factor 2 (Nrf2), and cytoprotective enzymes [such as γ-glutamate-cysteine ligase, NAD(P)H quinone oxidoreductase-1, and heme oxygenase-1 (γ-GCL, NQO1, and HO-1, respectively)]. However, whether Sirt1, Nrf2, and/or cytoprotective enzymes play a role in mitochondrial function and redox biology remains unclear. This figure was created by utilizing an image obtained from Servier Medical Art, licensed under a Creative Commons Attribution 4.0 Unported License (https://creativecommons.org/licenses/by/4.0/).

The evidence summarized in Table 1 and in Figure 6 highlights the multifaceted actions of DMF on mitochondrial function and redox biology, with implications for neuroprotection and the treatment of neurodegenerative disorders. Early cellular studies, such as those in dopaminergic CATH. a neurons exposed to tetrahydrobiopterin [BH4, which undergoes autoxidation and enhances the rate of dopamine oxidation and the formation of quinone proteins, which are toxic to brain cells by a mitochondria-dependent manner (Berman and Hastings, 1999; Jana et al., 2011)] demonstrated that DMF enhances respiratory chain activity, particularly Complexes I and IV, while suppressing cytochrome c release (Choi et al., 2006). These findings suggest a dual role in preserving bioenergetic output and preventing apoptosis. The similarity of the effects of DMF with those of N-acetylcysteine (NAC) raises the possibility that its protective mechanism involves redox-sensitive signaling, possibly mediated by GSH metabolism. However, these results are limited by the acute exposure design, which may not fully capture the dynamics of chronic neurodegenerative processes. Moreover, it can be speculated that DMF promoted mitochondrial protection by a mechanism associated with the upregulation of NQO1, a quinone reductase that can metabolize toxic quinones into less reactive agents (Ross and Siegel, 2021). Nonetheless, future research should combine NQO1 knock down with quinone flux analysis to confirm this speculation.

**TABLE 1:** The effects of DMF on mitochondrial function and redox biology.

Biological target	Experimental model	Major effects	References
Mouse dopaminergic CATH.a cells	DMF at 10 µM for 6 h before the administration of BH4 at 100 µM	Increased complexes I and IV activity; suppressed cytochrome c release; similar effects were induced by NAC	Choi et al. (2006)
Human neuroblastoma SK-N-SH cells exhibiting stable transfection of full-length ataxin-3 protein with 78 CAG repeats (MJD78 cells)	DMF at 10 µM for 24 h	Stimulated O_2_ flux, ATP-linked respiration, and maximum uncoupled capacity; diminished mitochondrial O_2_^−^⋅ production; upregulated Nrf2 and NQO1; JM17 (curcumin analog) induced similar effects at lower concentrations (0.3–1 µM) regarding mitochondrial function and redox biology and Nrf2 signaling	Wu et al. (2022)
iNeurons derived from *C9orf72* patient	DMF at 3–10 µM for 24 h	Decreased mitochondrial production of reactive species; upregulated Nrf2	Au et al. (2024)
Fibroblasts of patients with Friedreich’s ataxia	DMF at 30 µM for 24 h	Stimulated expression and immunocontent of Nrf2 and NQO1; increased expression of γ-GCL and HO-1; increased GSH levels; promoted frataxin expression; DMF did not modulate DJ-1	Petrillo et al. (2019)
Mice model of Friedreich’s ataxia	Model 1: DMF up to 160 mg/kg.day ^−^ ^1^ for 14 days, i.p. injection Model 2: DMF up to 160 mg/kg.day ^−^ ^1^ for 14 days, oral administration Model 3: DMF at 110 mg/kg.day ^−^ ^1^ for 14 days, oral administration Model 4: DMF at 110 mg/kg.day ^−^ ^1^ for 14 days, oral administration to FXNKD mice	Model 1: increased the immunocontent of frataxin and Cox4 subunit in mice liver Model 2: augmented aconitase activity; enhanced frataxin and Cox4 immunocontents in mice brain Stimulated aconitase activity in mice quadriceps DMF did not alter mtDNA copy number Model 3: Increased frataxin and cytochrome c oxidase levels in mice quadriceps Model 4: Stimulated aconitase, Complex II, and Complex IV activity; augmented mtDNA copy number in mice brain DMF failed to alter cytochrome c in mice quadriceps Increased frataxin in mice brain; enhanced frataxin and cytochrome c in mice cerebellum	Hui et al. (2021)
Spinal cord of *Abcd1* ^−^ mice	DMF at 100 mg/kg day ^−^ ^1^ for 4 months, oral administration	Upregulated Nrf2; reduced oxidative stress markers levels; stimulated the expression of Sirt1, Ppargc1a, Nrf1, and Tfam; increased mtDNA copy number; stimulated ATP production; attenuated the expression of Nfkb2, Il6, Tnfa, Ccl5, Cxcl10, and Ccr6	Ranea-Robles et al. (2018)

Studies using disease-relevant models further advance the understanding of the mechanisms induced by DMF. In Machado-Joseph disease (MJD) neuroblastoma cells, DMF improved O_2_ flux, ATP-linked respiration, and maximal uncoupled capacity while reducing mitochondrial superoxide production (Wu et al., 2022). These improvements were accompanied by upregulation of the Nrf2/NQO-1 pathway, reinforcing the notion that DMF acts as a pharmacological activator of antioxidant defenses. Yet, the comparison with the curcumin analog JM17, which exerted stronger antioxidant effects at lower concentrations, suggests that DMF may not be the most potent modulator of mitochondrial oxidative stress, highlighting the need for structure-activity studies to optimize therapeutic analogs.

Patient-derived iNeurons carrying *C9orf72* mutations, a model of ALS and frontotemporal dementia, responded to DMF with reduced mitochondrial reactive oxygen species (mtROS) and upregulation of Nrf2 (Au et al., 2024). While these findings align with previous results, they leave unresolved whether Nrf2 activation is causal or correlative in mitochondrial protection. The absence of functional bioenergetic assessments in this study limits its interpretability, particularly since redox improvement does not always equate to enhanced mitochondrial output.

In fibroblasts from Friedreich’s ataxia patients, DMF not only increased Nrf2, NQO-1, and GSH-related enzymes but also induced frataxin expression (Petrillo et al., 2019). The restoration of frataxin, a key protein in mitochondrial iron-sulfur cluster assembly (Campbell C. J. et al., 2021), suggests a unique disease-modifying potential of DMF in this context. Importantly, DJ-1 (Parkinsonism associated deglycase, also known as PARK7), another redox-sensitive protein whose levels are decreased in Friedreich’s Ataxia and contribute to a defective Nrf2-dependent signaling (Smith and Kosman, 2020), remained unaffected, indicating that protective scope stimulated by DMF may be selective rather than global across redox modulators. Nonetheless, the study lacked direct measurements of respiratory chain function, leaving unclear whether frataxin induction translates into functional mitochondrial recovery.

Animal models of Friedreich’s ataxia further support the benefits caused by DMF, with treatment increasing frataxin, aconitase activity, and respiratory chain components in multiple tissues (Hui et al., 2021). Interestingly, while some models showed increases in mitochondrial DNA (mtDNA) copy number, others did not, suggesting a context-dependent regulation of mitochondrial biogenesis. Moreover, the failure to consistently modulate cytochrome c levels across tissues underscores that the effects promoted by DMF are not uniform. This variability raises important questions about tissue-specific responses, which may have therapeutic implications given the differential vulnerability of brain regions in neurodegenerative diseases.

In a model of X-linked adrenoleukodystrophy (Abcd1^−/−^ mice), DMF exhibited a broader scope of action, not only upregulating Nrf2 and mitochondrial biogenesis-related genes (Sirt1, Ppargc1a, Nrf1, Tfam) but also increasing ATP production and attenuating inflammatory gene expression (Ranea-Robles et al., 2018). This dual effect on mitochondrial protection and inflammation is particularly relevant, as neurodegenerative disorders are increasingly recognized as disorders of immunometabolic dysfunction (Lonkar et al., 2025). However, it remains unclear whether mitochondrial preservation drives anti-inflammatory effects or whether both outcomes occur in parallel through independent signaling pathways.

Across acute toxicological insults and chronic degenerative milieus, DMF has been consistently associated with improvements in cellular redox balance and selected mitochondrial-related parameters, alongside modulatory effects on inflammatory gene networks. While activation of the Nrf2 pathway represents a well-characterized mechanism in several experimental settings, its direct involvement is not uniformly demonstrated across all models, and in many cases mitochondrial functional endpoints are only partially assessed. Accordingly, reported effects of DMF on mitochondrial homeostasis often rely on indirect redox-sensitive readouts or transcriptional changes rather than comprehensive bioenergetic or dynamic measurements. In this context, auxiliary pathways, including PGC-1α-, SIRT1-, and antioxidant enzyme–related networks (*e.g.*, HO-1 and glutathione metabolism), may contribute to the observed phenotypes in a model- and tissue-dependent manner. The heterogeneity of experimental designs and outcome measures underscores the need for more integrative approaches to delineate whether these pathways operate in parallel, sequentially, or independently of canonical Nrf2 signaling.

### 4.2 Effects of DMF on mitochondrial biogenesis and mitophagy

**FIGURE 7:**
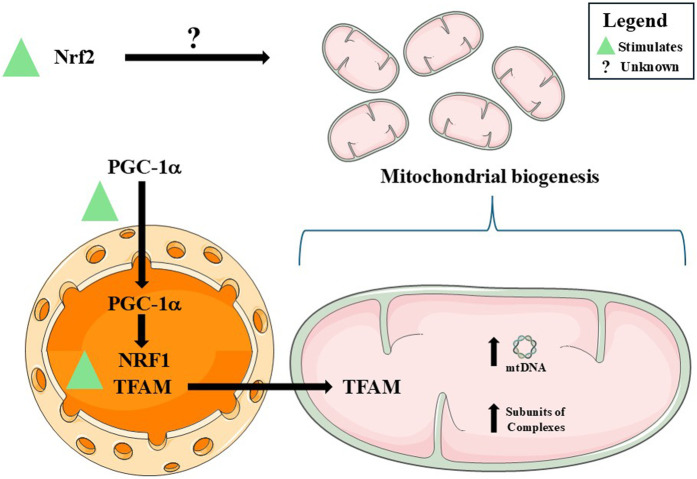
A summary of the effects induced by dimethyl fumarate (DMF) on mitochondrial biogenesis-related parameters. DMF stimulates (green triangle) peroxisome proliferator-activated receptor gamma coactivator 1-α (PGC-1α), that translocates to the nucleus of the cells and promotes nuclear respiratory factor 1 (NRF1) expression. NRF1, together with other regulatory agents, induces the expression of mitochondrial transcription factor A (TFAM), which in turn migrates to mitochondria and promotes mitochondrial DNA (mtDNA) synthesis, indicating mitochondrial biogenesis. DMF also upregulated the expression of subunits of the Complexes of the oxidative phosphorylation (OXPHOS) system. Nuclear factor erythroid 2-related factor 2 (Nrf2) stimulation by DMF also plays an important role in the induction of mitochondrial biogenesis, although it is not yet completely understood. Please, read the text for details. This figure was created by utilizing images obtained from Servier Medical Art, licensed under a Creative Commons Attribution 4.0 Unported License (https://creativecommons.org/licenses/by/4.0/).

Across neural and peripheral systems, DMF is recurrently associated with the activation of redox-sensitive transcriptional programs that include gene networks classically linked to mitochondrial biogenesis and, in specific contexts, to quality control (Figure 7; Table 2). Rather than uniformly inducing demonstrable expansion of mitochondrial mass, turnover, or function, these responses more consistently reflect engagement of upstream regulatory circuits (*i.e.*, transcriptional or copy-number readouts), most notably those centered on Nrf2, that are commonly interpreted as preparatory or permissive for mitochondrial adaptation. This coherence, therefore, becomes appreciable only when disparate experimental models are interpreted along a shared interpretative framework, rather than a fully unified mechanism.

**TABLE 2:** The effects of DMF on mitochondrial biogenesis and mitophagy.

Biological target	Experimental model	Major effects	References
Rat oligodendrocyte progenitor cells	DMF at 10 µM for 1–24 h	Increased O_2_^−^⋅ mitochondrial production; decreased MMP; upregulated Nrf2; upregulated PGC-1α, TFAM, and COX1; upregulated Mn-SOD and HO-1; increased mitochondrial mass	De Nuccio et al. (2020)
Healthy human fibroblast cell line AG09429, C57BL/6 mice skeletal muscle and cerebellum, and PBMCs collected from patients suffering from multiple sclerosis	*In vitro*: DMF at 30 μM, for 48 h *In vivo*: DMF at 10 mg/kg, once a day for 2 weeks, i.p. injection PBMCs: patients were administrated with DMF for 3 months (DMF dose not mentioned in the work)	*In vitro*: enhanced mtDNA copy number; stimulated the expression of TFAM, MT-ND2, MT-ND6, SDHA, SDHB, MT-CYB, CYC1, MT-CO1, MT-CO2, ATP5B, and MT-ATP6; increased OCR; mitochondrial biogenesis-related effects dependent on Nrf2 *In vivo*: increased mtDNA copy number; stimulated the expression of MT-CO1, MT-ATP6, and MT-ND2 PBMCs: enhanced mtDNA copy number; stimulated the expression of MT-ND6, MT-CYB, MT-CO2, and MT-ATP6	Hayashi et al. (2017)
Rat spinal cord	DMF at 300 mg/kg, once a day for 5 days beginning from day 14 of the induction of spinal cord lesion by MIA at 1 mg/50 µL	Increased the immunocontents of PGC-1α, NRF1, TFAM, and Nrf2; enhanced mtDNA copy number; mitochondrial biogenesis-related effects were dependent on Nrf2	Gao et al. (2022)
Several brain regions of aged C57Bl/6 mice	DMF at 10 mg/kg.day ^−^ ^1^ for 50 days, dissolved in water	Increased mtDNA copy number; reduced damaged mtDNA levels; stimulated the expression of Sirt1, Nrf2, and SOD2 (gene coding for Mn-SOD)	Sadovnikova et al. (2021)
Mid-brain of C57BL/6 mice administrated with MPP^+^	DMF at 15–60 mg/kg.day ^−^ ^1^ for 21 days beginning 7 days after induction of lesion	Increased the immunocontents of Nrf2, PINK1, Parkin, BNIP3, and Beclin1; reduced the immunocontents of LC3A/BII and BCL-2; DMF did not alter NRF1	Pinjala et al. (2024)
Several organs of Yorkshire piglets	DMF at 30 mg/kg.day ^−^ ^1^ for 4 days in an experimental model of IHCA	Cerebral cortex: upregulated subunits of the complexes I, IV, and V; increased the levels of proteins involved in the control of mitochondrial morphology; failed to restore mitochondrial function (did not alter Complex I-II activity)	Piel et al. (2024)
Lymphoblasts and fibroblasts obtained from patients suffering from Friedreich’s ataxia	DMF at 10–30 μM, for 48 h	Stimulated frataxin expression; Increased mtDNA copy number	Jasoliya et al. (2019)
Fibroblasts obtained from patients suffering from Friederich’s ataxia (GM-4078 cell line) and YG8LR mice model of Friederich’s ataxia	*In vitro*: DMF at 30 µM for 48 h *In vivo*: 10 mg/kg.day ^−^ ^1^ each for 5 days	*In vitro*: stimulated frataxin expression; increased mtDNA copy number *In vivo*: DMF did not alter frataxin expression and mtDNA copy number	Abeti et al. (2022)

For instance, in rat oligodendrocyte progenitors, DMF elicited a transient pro-oxidant pulse, characterized by increased mitochondrial O_2_^−•^ production and reduced MMP, followed by induction of Nrf2, PGC-1α, TFAM, COX1, Mn-SOD, and HO-1 (De Nuccio et al., 2020). The fact that 4-HT (Mn-SOD mimetic) and ZnPP-IX (HO-1 inhibitor) attenuate cytoprotection suggests that low-level oxidative cues are not collateral stress but may function as instructional signals that engage redox-sensitive transcriptional regulators linked to mitochondrial adaptation, rather than unequivocal drivers of mitochondrial biogenesis *per se*. While this coordinated gene induction is compatible with activation of a biogenic program, the available data primarily substantiate transcriptional engagement rather than direct increases in mitochondrial content or sustained bioenergetic capacity. Such a signaling-based interpretation aligns with the established sensitivity of oligodendroglial cells to redox fluctuations during differentiation and myelin maintenance (Magalhães et al., 2017; Narine and Colognato, 2022).

When extended to human fibroblasts, mouse skeletal muscle and cerebellum, and peripheral blood mononuclear cells (PBCMs) from patients with multiple sclerosis, DMF treatment is similarly associated with increased mtDNA copy number, coordinated induction of nuclear- and mitochondrial-encoded genes (including TFAM, MT-ND2/ND6, SDHA/B, MT-CYB, MT-CO1/2, ATP5B, and MT-ATP6), and enhanced O_2_ consumption (Hayashi et al., 2017). Collectively, these endpoints are frequently interpreted as evidence of mitochondrial biogenesis; however, in the absence of ultrastructural quantification (*e.g.*, transmission electron microscopy - TEM) or direct assessment of mitochondrial protein synthesis, they are more conservatively viewed as reflecting transcriptional and genomic remodeling. Such changes may represent mitochondrial priming or compensatory gene-expression responses rather than unequivocal expansion of the mitochondrial network.

A similar pattern emerges in the monosodium iodoacetate (MIA)-induced spinal cord injury model, in which DMF increased PGC-1α, NRF1, TFAM, and mtDNA in an Nrf2-dependent manner (Gao et al., 2022). These findings indicate that the transcriptional architecture classically associated with mitochondrial biogenesis remains responsive, even under inflammatory and metabolic stress. Whether these transcriptional changes translate into sustained increases in mitochondrial content or functional throughput under physiological load, however, remains unresolved. Given that Nrf2 regulates HO-1, an enzyme implicated in both mitochondrial signaling (Hull et al., 2016; Navarro et al., 2017) and anti-inflammatory responses (Campbell N. K. et al., 2021), clarifying the role of the Nrf2/HO-1 axis becomes not merely an adjunct question but a necessary step in determining whether DMF’s effects are mechanistically unified across neural injury paradigms.

A similar integrative perspective bridges findings from aging-related paradigms, DMF increased mtDNA copy number, reduced damaged mtDNA, and activated SIRT1, Nrf2, and SOD2 across multiple brain regions (Sadovnikova et al., 2021). Rather than directly establishing *de novo* mitochondrial biogenesis, these outcomes support a role for DMF in reinforcing mitochondrial genome integrity and antioxidant defenses. Such effects are consistent with modulation of mitochondrial maintenance and fidelity, processes that may indirectly favor functional resilience without necessarily increasing mitochondrial abundance. Yet, without functional assays (*e.g.*, respiration, coupling efficiency, and ROS yield), such structural and genomic improvements remain inferential; thus, morphological quantification and performance testing remain indispensable to confirm mitochondrial biogenesis.

Insights into mitochondrial turnover arise from dopaminergic injury models, where DMF upregulated Nrf2, PINK1, Parkin, BNIP3, Beclin-1, LC3A/B-II, and BCL-2 in the absence of NRF1 induction (Pinjala et al., 2024). This expression profile is compatible with preferential engagement of signaling nodes associated with mitophagy-related pathways rather than coordinated activation of canonical biogenic machinery, *i.e.*, initial removal of dysfunctional mitochondria may precede or condition any subsequent biogenic response (mitochondrial re-population). However, without time-resolved analyses or direct measurements of mitophagic flux, these molecular changes are more appropriately interpreted as modulation of mitophagy-associated signaling rather than confirmation of enhanced mitochondrial turnover.

The dissociation between transcriptional or proteomic remodeling and functional recovery is particularly evident in severe stress models. In piglet cortex following in-hospital cardiac arrest (IHCA), DMF increased subunits of mitochondrial respiratory complexes and morphology-related proteins without restoring mitochondrial function (Piel et al., 2024). In contrast, porcine cardiac tissue from the same paradigm exhibited improvements in mitochondrial network organization and ultrastructural features. This dissociation underscores that proteomic enrichment of mitochondrial components does not necessarily equate to functional recovery, particularly under conditions of severe energetic collapse.

Translational complexity is further underscored by studies in Friedreich’s ataxia. In patient-derived cells, DMF increases frataxin expression and mtDNA content (Jasoliya et al., 2019), findings that are consistent with transcriptional or genomic modulation but insufficient to confirm induction of mitochondrial biogenesis or involvement of Nrf2 signaling. However, short-term *in vivo* treatment fails to reproduce these effects in YG8LR mice (Abeti et al., 2022), highlighting the influence of pharmacokinetics, tissue context, and temporal dynamics on the engagement of nuclear-encoded mitochondrial programs.

Taken together, these heterogeneous findings support an interpretative framework in which DMF preferentially engages transcriptional and signaling programs associated with mitochondrial biogenesis, antioxidant defense, and turnover in a context-dependent manner. Yet functional rescue remains variable across models, emphasizing that durable neurological benefit will depend on mechanistic clarification, exposure optimization, and adoption of rigorous flux-based methodologies capable of distinguishing molecular signatures from true mitochondrial recovery. Progression beyond priming toward fully realized mitochondrial remodeling appears variable and frequently unverified by direct functional or structural criteria, underscoring the need for integrative, flux-based validation strategies.

Considering the conceptual continuity that emerges across these diverse models, future research priorities can be articulated in an equally integrated manner. Multi-omics profiling should be coupled with ultrastructural analyses (*e.g.*, TEM) to differentiate genuine mitochondrial biogenesis from transcriptional priming. Mechanistic causality should be tested through genetic (*e.g.*, CRISPR-Cas9 knockouts) or pharmacologic disruption of Nrf2, HO-1, and SIRT1, thereby elucidating whether the shared signatures observed across tissues indeed converge upon a unified regulatory axis. Moreover, defining DMF’s hormetic window through dose-time matrices quantifying ROS dynamics, MMP fluctuations, and downstream transcription would anchor mitochondrial adaptation within measurable signaling thresholds. Flux-level assays, including mt-Keima or mito-QC for mitophagy, MitoTimer for turnover, ^35S^-methionine or puromycin labeling for nascent mitochondrial translation, and TFAM-mtDNA occupancy (ChIP), should replace static proxies wherever possible. Complementary high-resolution respirometry under substrate-inhibitor titrations will further resolve how DMF remodels ATP production, proton leak, and redox balance.

Finally, *in vivo* studies must incorporate longitudinal designs, BBB-exposure measurements, and cell-type–resolved analyses, especially given the broad preclinical dose range (10–300 mg kg^−1^). Bayesian pharmacokinetic modeling, informed by fumarate hydrolysis and distribution kinetics, may refine dose–response extrapolation to humans. Comparative analyses of DMF, MMF, and next-generation fumarate esters may ultimately reveal whether mitochondrial benefits and tolerability can be decoupled or optimized for distinct diseases such as MS, PD, ischemia-reperfusion injury, and FA.

Building on the evidence that DMF reshapes mitochondrial quantity and quality through tightly regulated programs of biogenesis and turnover, a broader conceptual role for mitochondria emerges. Rather than acting as passive targets of stress, mitochondria function as integrative signaling hubs that decode redox, metabolic, and inflammatory cues into adaptive cellular decisions. Transient oxidative signals are not merely buffered but translated into coordinated transcriptional, structural, and quality-control adjustments that recalibrate mitochondrial performance under stress. Crucially, these adaptive programs position mitochondria at the crossroads between survival and death. The same organelle network that expands or renews itself to sustain bioenergetic and redox homeostasis also harbors the molecular machinery governing mitochondrial outer membrane permeabilization, cytochrome c release, and caspase activation. Thus, mitochondrial remodeling and apoptotic control are not separable phenomena but interdependent layers of a unified stress-response architecture. Within this framework, mitochondrial signaling emerges not only as a determinant of cellular resilience, but also as a decisive regulator of intrinsic apoptotic pathways, directly linking adaptive mitochondrial states to the molecular execution of cell death.

### 4.3 Effects of DMF on mitochondria-associated anti-apoptotic effects

**FIGURE 8:**
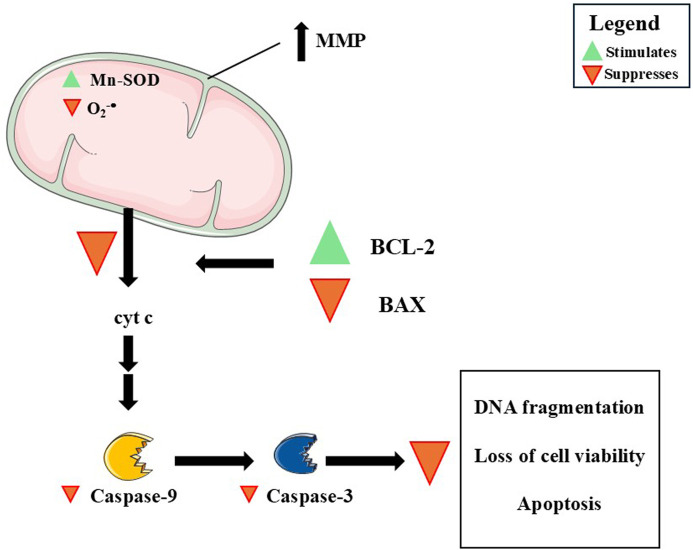
A summary of the mitochondria-related anti-apoptotic effects induced by dimethyl fumarate (DMF). The DMF-induced stimulation of B-cell lymphoma 2 (BCL-2; green triangle), together with the observed downregulation of BCL2-associated X protein (BAX; red triangle), favors the maintenance of intramitochondrial cytochrome c (cyt c) levels and mitochondrial membrane potential (MMP). Inhibition of cyt c release causes a decrease in the activation of caspases-9 and -3, which would mediate pro-apoptotic actions. The stimulation of manganese-dependent superoxide dismutase (Mn-SOD) and the decrease in superoxide radical (O_2_^−•^) production promoted by DMF may also play a role in its anti-apoptotic action, as it reduces the chances of oxidation of cardiolipin, a lipid that keeps cyt c associated with the inner mitochondrial membrane. Please, see the text for more details on the mechanisms of action related to the anti-apoptotic role of DMF. This figure was created by utilizing images obtained from Servier Medical Art, licensed under a Creative Commons Attribution 4.0 Unported License (https://creativecommons.org/licenses/by/4.0/).

As observed across neural and peripheral models, DMF consistently attenuates mitochondria-dependent apoptosis by stabilizing mitochondrial function and reprogramming intrinsic death checkpoints. At the molecular level, this protection is reflected in preservation of mitochondrial membrane potential, rebalancing of BCL-2 family proteins, limitation of cytochrome c release, and suppression of downstream caspase activation. These effects position mitochondria not merely as targets of injury, but as active arbiters of whether stressed cells undergo recovery or engage the intrinsic apoptotic cascade (Figure 8; Table 3).

**TABLE 3:** The effects of DMF on mitochondria-associated anti-apoptotic effects.

Biological target	Experimental model	Major effects	References
158 N murine oligodendrocytes	DMF at 1–50 µM for 24 h after the administration of 7-ketocholesterol at 25–50 µM for further 24 h	Attenuated loss of cell viability and MMP collapse; decreased production of O_2_^−^⋅ and H_2_O_2_; downregulated PARP; suppressed caspase-3 activation; reduced nuclear DNA fragmentation	Zarrouk et al. (2017)
158 N murine oligodendrocytes	DMF at 25 µM in the presence of 7β-hydroxycholesterol at 50 µM for 24 h	Attenuated oxidative stress; increased SOD, CAT, and GPx activity; enhanced cardiolipin levels; decreased mitochondrial O_2_^−^⋅ production; attenuated mitochondrial fragmentation; abrogated caspase-3 activation; diminished LC3-II/LC3-I ratio; increased levels of malic acid and citric acid	Sghaier et al. (2019)
Human dopaminergic SH-SY5Y cell line	DMF at 30 µM for 2 h prior to the administration of Aβ_1-42_ at 1 μM for 24 h	Failed to downregulate p38 and JNK signaling pathways; upregulated Akt; blocked cytochrome c release; decreased BAX levels; suppressed caspase-9 and caspase-3 activation	Rajput et al. (2020)
Mice spinal cord tissue	DMF at 30 mg/kg, i.p. injection, 1 and 6 h after the induction of SCI	Upregulated BNDF, GDNF, and NT-3; downregulated MPO, IL-1β, TNF-α, COX-2, and iNOS; downregulated AIF and Fas ligand; upregulated BCL-2, Nrf2, HO-1, GPx-1, and Mn-SOD	Cordaro et al. (2017)
Rat brain and kidney	DMF at 12.5–50 mg/kg once a day for 6 weeks after induction of TBI	Reduced BAX levels; increased BCL-2 levels; decreased the number of apoptotic cells; upregulated Nrf2 and HO-1; downregulated IL-1β	Gao et al. (2024)
Rat hippocampus	DMF at 60 mg/kg.day ^−^ ^1^ for 10 weeks, oral administration, 1 h prior to the administration of pentylenetetrazol (PTZ)	Decreased caspase-3 expression; DMF did not alter BAX and BCL-2 expression or immunocontent	Singh et al. (2019)
Mice brain	DMF at 15–60 mg/kg.day ^−^ ^1^ for 21 days simultaneously to the administration of rotenone at 1.5 mg/kg (i.p. injection)	Decreased α-synuclein content; upregulated tyrosine hydroxylase; increased Nrf2 levels; reduced the immunocontent of p53, BAX, cleaved caspase-3, and cathepsin D; upregulated BCL-2; upregulated TIGAR and LAMP2	Khot et al. (2023)
Rat hippocampus	DMF at 100 mg/kg, orally administrated three times per week during 4 weeks after the induction of CCH	Decreased BAX levels; decreased caspase-3 activity; upregulated BCL-2, Nrf2, NQO1, and HO-1; downregulated NF-κB and TNF-α levels; inhibition of Nrf2 blocked the effects on NQO1, NF-κB, and TNF-α	Shavakandi et al. (2022)
Preconditioned mesenchymal stem cells (MSC) administrated to rat hippocampus	MSC were treated with DMT at 20 µM and administrated to the hippocampus of rats exposed to Aβ_1-42_	Stimulated BDNF and NGF expression; decreased the expression of BAX, caspase-3, and cytochrome c; enhanced BCL-2 expression	Babaei et al. (2023)

In 158N oligodendrocytes challenged with oxysterols, DMF stabilized MMP, curtailed O_2_^−•^ and H_2_O_2_ production, limited poly (ADP-ribose) polymerase (PARP) and caspase-3 activation, and reduced nuclear fragmentation (Zarrouk et al., 2017; Sghaier et al., 2019). In human dopaminergic SH-SY5Y cells exposed to Aβ_1-42_, DMF stimulated Akt signaling, restrained cytochrome c release, downregulated BAX, and blocked caspases-9 and -3, despite minimal impact on stress kinases p38 and c-Jun N-terminal kinase (JNK), underscoring pathway selectivity upstream of mitochondria (Rajput et al., 2020). These findings highlight selective modulation of mitochondrial checkpoints upstream of executioner caspases. *In vivo*, DMF lessened apoptotic signaling after spinal cord injury (SCI) (Cordaro et al., 2017), traumatic brain injury (TBI) (Gao et al., 2024), pentylenetetrazol (PTZ)-induced seizures (Singh et al., 2019), rotenone toxicity (Khot et al., 2023), and chronic cerebral hypoperfusion (CCH) (Shavakandi et al., 2022), with convergent decreases in BAX, cleaved caspase-3, AIF, and Fas ligand, and increases in BCL-2, collectively indicating modulation of intrinsic apoptotic thresholds.

Redox regulation emerges as a central modulatory theme. DMF stimulated antioxidant defenses (SOD, CAT, GPx-1, and Mn-SOD), restores cardiolipin, and reduces mitochondrial O_2_^−•^ generation, thereby limiting MOMP and caspase engagement (Sghaier et al., 2019; Cordaro et al., 2017). The increase in cardiolipin is mechanistically notable because it anchors respiratory chain complexes and modulates cytochrome c mobilization, offering a direct route to constrain apoptosis (Li et al., 2025). Contextual variability remains evident, however, as DMF reduces the LC3-II/LC3-I ratio and mitochondrial fragmentation in oxysterol-treated oligodendrocytes, a pattern compatible with suppression of autophagy- or mitophagy-associated signaling under acute oxidative stress (Sghaier et al., 2019). Such suppression could be protective during acute oxidative insults but might prove maladaptive if damaged mitochondria require clearance, highlighting the need for precise temporal mapping of mitophagy flux.

Nrf2 signaling repeatedly emerges as a central, though not exclusive, modulatory axis linking redox balance, inflammation, and mitochondrial survival. DMF-induced increases in Nrf2 and downstream effectors such as HO-1 and NQO-1 are accompanied by suppression of NF-κB-dependent cytokines (TNF-α, IL-1β) and inflammatory mediators [myeloperoxidase (MPO)/iNOS] (Cordaro et al., 2017; Gao et al., 2024; Shavakandi et al., 2022). Inhibition of Nrf2 nuclear translocation abrogates several of these effects (Shavakandi et al., 2022), supporting a contributory role for this pathway. Nonetheless, Nrf2 signaling should be viewed as a central modulatory axis rather than a singular causal determinant of mitochondrial protection. Additional associations with TP53-induced glycolysis and apoptosis regulator (TIGAR) and lysosome-associated membrane protein 2 (LAMP2) expression (Khot et al., 2023), as well as increased neurotrophic factor [brain-derived neurotrophic factor (BDNF), glial cell line-derived neurotrophic factor (GDNF), neurotrophin-3 (NT-3)] levels and benefits from DMF-preconditioned mesenchymal stem cells (Cordaro et al., 2017; Babaei et al., 2023), further suggest coordinated modulation of metabolic, lysosomal, and survival pathways that converge on mitochondrial stability.

Despite these convergent trends, substantial heterogeneity persists across models with respect to dose (1–50 µM *in vitro*; 12.5–100 mg/kg *in vivo*), timing (pretreatment vs. post-injury), cell type (oligodendrocytes vs. dopaminergic-like vs. mixed brain regions), and outcome measures. For instance, in hippocampal PTZ experiments, executioner caspase activity was reduced without clear shifts in upstream BCL-2 family balance (Singh et al., 2019), whereas in others (SH-SY5Y cells) mitochondrial protection occurs independently of stress-kinase modulation (Rajput et al., 2020). These differences emphasize that DMF biases intrinsic apoptotic thresholds through multiple, context-dependent nodes rather than through a single invariant mechanism.

Collectively, the available evidence positions DMF as a potent modulator of mitochondria-associated apoptotic signaling in neural systems, acting through integrated redox, inflammatory, and intrinsic death-regulatory pathways. While the protective signature is compelling, precise delineation of causal mechanisms and temporal dynamics remains essential to optimize therapeutic translation in neurodegenerative and acute CNS injury settings.

Regarding future investigations, rigorous causality tests are needed, such as genetic Nrf2 (and Keap1) loss-of-function across neural subtypes, live-cell MOMP imaging and cardiolipin tracking, and true mitophagy-flux assays (*e.g.*, mito-Keima) to define when DMF preserves or impairs mitochondrial quality control. Mechanistic dissection of TIGAR and LAMP2 as mediators rather than correlates, and of the role of Akt in blocking cytochrome c release, is warranted. Region-resolved *in vivo* studies combining neuroinflammation readouts with mitochondrial endpoints, alongside pharmacokinetics/brain penetration and dose–response mapping, will sharpen translational predictions. Finally, testing DMF with pro-mitophagic or neurotrophic co-therapies may reconcile protection with necessary mitochondrial turnover.

### 4.4 Impairing mitochondrial function with DMF as a therapeutic strategy in neurological disorders

**FIGURE 9:**
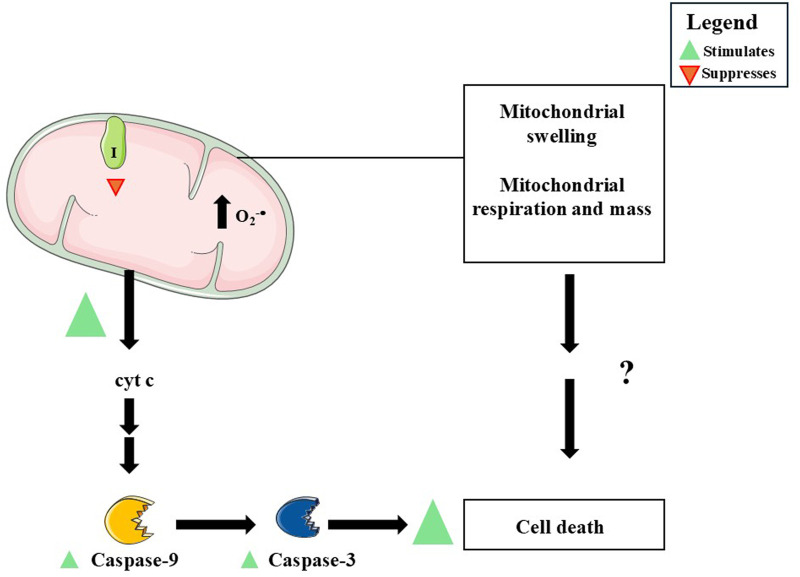
A summary of the mechanisms of action by which dimethyl fumarate (DMF) induces mitochondrial dysfunction of pharmacological interest. DMF induces a decrease (red triangle) in the activity of Complex I, in mitochondrial respiration, and in the glycolytic rate (please, see the text for details). Increased mitochondrial superoxide radical (O_2_^−•^) production and cytochrome c release, followed by activation of pro-apoptotic caspases, have been observed in different cell types. Changes in mitochondrial mass and architecture, such as organelle swelling, have also been reported in cells exposed to DMF. It remains to be clarified whether the decrease in mitochondrial respiration and changes in the mass and architecture of these organelles play a role in the induction of cell death in cells exposed to DMF. This figure was created by utilizing images obtained from Servier Medical Art, licensed under a Creative Commons Attribution 4.0 Unported License (https://creativecommons.org/licenses/by/4.0/).

DMF demonstrates how controlled mitochondrial dysfunction can be therapeutically harnessed to reshape immune cell fate. In RRMS, clinical benefit correlates with a mitochondria centric shift in T cell metabolism: routine dosing decreases relapse frequency while dampening oxidative phosphorylation (Gold et al., 2012; Liebmann et al., 2021). High resolution respirometry in patient derived CD4^+^ and CD8^+^ lymphocytes showed that DMF lowers basal and maximal respiratory capacity, collapses spare glycolytic reserve, and remodels cristae architecture, culminating in cytochrome c release and caspase −9 and −3 activation (Liebmann et al., 2021) (Figure 9; Table 4).

**TABLE 4:** Impairing mitochondrial function with DMF as a therapeutic strategy.

Biological target	Experimental model	Major effects	References
CD4^+^ and CD8^+^ T-cells obtained from patients suffering from RRMS and from healthy subjects	DMF treatment as follows - week 0: 120 mg.day-1; week 1: 120 mg twice daily; week 2: 240 mg in the morning and 120 mg in the evening; week 3: 240 mg twice daily; weeks 4 and 24: 240 mg twice daily	RRMS patients: increased apoptotic rate; enhanced caspase-9 and caspase-3 levels; reduced mitochondrial respiratory capacity; decreased glycolytic rate Healthy subjects: decreased GSH levels; stimulated total and mitochondrial production of O_2_^−•^; increased MMP; decreased mitochondrial respiration and mass; induced mitochondrial swelling; promoted cytochrome c release; increased caspase-9 and caspase-3 levels Co-administration of GSH, NAC or mitoQ attenuated the effects induced by DMF on mitochondria-related parameters	Liebmann et al. (2021)
Human microglial clone 3 cell line (HMC3)	DMF at 50 µM for 24 h in the presence or absence of LPS at 1 μg/mL	Downregulated IL-1β, IL-6, TNF-α, and iNOS; decreased glycolytic rate; reduced mitochondrial respiration; diminished Complex I activity; failed to modulate the activity of the complexes II and V; decreased H_2_O_2_ production by mitochondria consuming pyruvate/malate; decreased MDA and HNE adducts levels in mitochondria	Sangineto et al. (2024)

Paradoxically, DMF hyperpolarized mitochondria while shrinking organelle mass, implying a compensatory tightening of the proton gradient despite curtailed electron flux. This redox imbalanced state amplified O_2_^−•^ production, yet it is readily reversed by GSH, NAC or mitoquinone mesylate (mitoQ). Rescue of redox poise not only reinstated oxidative phosphorylation but also blocked DMF induced lymphocyte quiescence, highlighting a critical role for redox and/or thiol dependent signaling (Zhang and Forman, 2012; Kukulage et al., 2022; Lin et al., 2024; Vašková et al., 2023). Besides, this data implicates mitochondrial oxidative damage as key mechanistic features of DMF toxicity in immune cells. Measuring GSH pools before therapy may therefore forecast patient sensitivity to DMF.

An analogous metabolic checkpoint operates in innate immunity. In LPS stimulated human microglial clone 3 cells, DMF suppressed iNOS and the pro-inflammatory cytokines IL-1β, IL-6 and TNF-α, aligning with its anti-inflammatory profile, while preventing the surge in both glycolytic flux and O_2_ consumption (Sangineto et al., 2024). This anti-inflammatory effect coincided with impaired mitochondrial respiration, reduced Complex I activity, and decreased reactive species production and lipid peroxidation markers, reflecting a broad dampening of mitochondrial bioenergetics. Notably, no compensatory activity was observed in Complexes II or V, suggesting a non-redundant impairment in mitochondrial electron transport chain function–effects that may be exploited to temper microglial overactivation in neurodegeneration.

Systems level evidence positions the transcription factor Nrf2 at the apex of this response. Beyond antioxidative gene induction, Nrf2 regulates mitochondrial biogenesis, fusion and mitophagy, thereby synchronizing organelle architecture with fluctuating energetic demand (Luchkova et al., 2024). Whether DMF signals through Nrf2 to orchestrate mitochondrial quality control alongside redox homeostasis remains a pressing mechanistic question.

Clinically, the lymphopenia occasionally observed during DMF therapy most likely reflects the same mitochondrial stress response that underpins efficacy (Dinoto et al., 2022). Stratification based on respiratory reserve, GSH/GSSG ratio or Nrf2 dependent gene signatures could predict benefit to risk ratios and guide adjunctive thiol supplementation. In neurodegenerative settings, carefully titrated DMF might recalibrate microglial metabolism without broad immunosuppression, offering neuroprotection through metabolic modulation.

In summary, DMF illustrates that calibrated impairment of mitochondrial bioenergetics can attenuate immune cells activation. In that regard, off-target effects on mitochondrial function merit careful consideration, since the redox-sensitive nature of mitochondria renders them particularly vulnerable to electrophilic stressors such as DMF, especially in non-pathological tissues. Future works should quantify drug induced shifts in mitochondrial GSH, delineate Nrf2 centric transcriptional circuits, and map complex specific respiratory changes across immune subsets, thereby refining the therapeutic window of DMF and inspiring novel mitochondria targeted interventions.

### 4.5 Mitochondria-associated cytotoxicity induced by DMF in brain cells

Martinez et al. (2024) reported the effects caused by DMF in mouse primary cerebral vascular smooth muscle cells, alone and with TNF-α, revealing a coordinated shift from mitochondrial to glycolytic ATP production, increased lactate output under inflammatory stimulus, heightened mitochondrial ROS, apoptosis, and glutathione depletion (higher GSSG/GSH) (Table 5). Notably, pharmacologic inhibition of Nrf2 blunts the glycolytic ATP increase, indicating that part of the metabolic rewiring is Nrf2-dependent. These findings align with an electrophile-driven mechanism in which DMF perturbs redox buffering, transiently activating Nrf2 yet simultaneously imposing mitochondrial impairment, manifesting as ROS elevation and apoptosis, particularly when inflammatory signaling is present. The convergence of redox imbalance (GSH consumption), mitochondrial dysfunction (impaired oxidative ATP synthesis, ROS production), and inflammatory cues (TNF-α-augmented glycolysis) supports a model of stress-adaptation failure, where Nrf2-mediated glycolytic compensation is insufficient to prevent cell death under combined insults.

**TABLE 5:** Mitochondria-associated cytotoxicity caused by DMF.

Biological target	Experimental model	Major effects	References
Mouse primary cerebral vascular smooth muscle cells	DMF at 10–50 µM in the presence of TNF-α at 50 ng/mL for up to 24 h	Decreased mitochondrial and increased glycolytic synthesis of ATP in a dose-dependent manner; Nrf2 inhibition suppressed the effects on the glycolytic synthesis of ATP; in the presence of TNF-α, stimulated glycolysis and augmented the production of lactate in an Nrf2-dependent manner; increased mitochondrial ROS production; stimulated apoptosis; decreased GSH levels (increased GSSG/GSH ratio)	Martinez et al. (2024)

Methodologically, the use of primary cerebrovascular smooth muscle cells strengthens physiological relevance for the neurovascular unit. Besides, translation requires caution, since exposure windows are acute, concentrations may exceed steady-state levels achieved *in vivo*, and outcomes were captured at the population level without single-cell resolution. The explicit link between mtROS and downstream apoptosis, while plausible, is correlative here and would benefit from targeted rescue experiments (*e.g.*, mitochondria-directed antioxidants or genetic modulation of mitochondrial quality control).

Future work includes, for example, *in vivo* validation in neuroinflammation models to test cerebrovascular toxicity and barrier integrity, metabolomics/fluxomics to map the Nrf2-dependent rerouting of carbon and nitrogen metabolism, causal tests of mtROS using site-specific ROS modulators and redox-insensitive Nrf2 constructs, dissection of mitochondrial dynamics and mitophagy as determinants of cell fate, and stratification of inflammatory contexts (*e.g.*, TNF-α *versus* mixed cytokines) to define interaction surfaces with DMF.

Together, these results position DMF as a dual-edged electrophile, since it triggers Nrf2-linked glycolytic compensation yet concurrently compromises mitochondrial function and redox homeostasis, tipping cells toward apoptosis under inflammatory stress. This mechanistic tension (adaptation *versus* injury) should guide risk-benefit assessments for DMF in neurovascular settings.

### 4.6 Future directions

According to the data previously published by different research groups, it is possible to make the following suggestions as future directions in research related to DMF-induced effects on the mitochondrial physiology of CNS cells:

- Therapeutic window mapping by cell type and inflammatory state. Implement dose–time–inflammation matrices across neurons, astrocytes, oligodendroglia, microglia, and cerebrovascular cells, integrating OCR and extracellular acidification rate, ATP production routes, and apoptosis/mitophagy flux assays (PINK1/Parkin, BNIP3/LC3 turnover). Co-administration with redox modulators should be systematically tested to define rescue ranges and liabilities.
- Mechanistic dissection of Nrf2-centered mitochondrial remodeling. Test causality for Nrf2, NQO1, and downstream pathways (Sirt1-PGC-1α-NRF1-TFAM) using genetic perturbations and pharmacologic inhibitors; resolve whether DMF triggers *bona fide* mitochondrial biogenesis *versus* selective turnover (mitophagy) followed by compensatory replication. Include ultrastructural and network-level metrics (TEM, super-resolution, mito-network topology).
- Function over form: link proteomic shifts to electron-transfer capacity. Pair complex subunit abundance with Complex I–IV enzymology, supercomplex assembly, cardiolipin integrity, and ROS site mapping to explain dissociations between biogenetic signatures and respiratory outcomes in stress models.
- Disease-model validation and patient stratification. Extend positive signals (*e.g.*, frataxin induction, reduced mtROS in C9orf72 iNeurons) into organoids and *in vivo* CNS models, incorporating pharmacokinetics/pharmacodynamics in brain and spinal cord. Evaluate gender, age, and disease-stage effects, and explore biomarkers (mtDNA copy number, NQO1 induction, TIGAR/LAMP2) to guide precision dosing.
- Immune–mitochondrial cross-talk. In microglia and T cells, delineate how the immunomodulation induced by DMF intersects with bioenergetics (glycolysis-OXPHOS coupling), and whether anti-inflammatory gains can be uncoupled from mitochondrial penalties *via* schedule or co-therapy optimization.

### 4.7 Conclusion

Clinical disappointments with generic scavengers (vitamins C/E) highlight a core lesson: CNS redox pathology is not a homogeneous excess of “free radicals” but a compartmental, pathway-specific breakdown involving mitochondria-immune crosstalk. Emerging directions (*e.g.*, mitochondria-targeted antioxidants, Nrf2 activation, modulation of NOX/NOS, nano-delivery, and gene/epigenetic interventions) aim to restore networked redox homeostasis rather than merely quench radicals. Therapeutics that reprogram the Nrf2 axis and mitochondrial resilience should be evaluated with attention to subcellular targeting, patient-specific redox signatures, and inflammatory tone, a precision-redox framework that aligns with current artificial intelligence/multi-omics strategies. DMF exerts a bidirectional influence on mitochondria in CNS-related systems: it frequently activates Nrf2-dependent programs that favor redox resilience, biogenesis markers, and anti-apoptotic signaling in neurons and oligodendroglia, yet it can blunt respiration or promote apoptosis in immune and vascular cells or under heightened inflammation. The totality of evidence argues for a nuanced, context-aware deployment of DMF in neurological disorders, anchored in cell type-specific dosing, stringent functional readouts, and, where appropriate, adjunctive redox buffering to preserve mitochondrial benefits while minimizing liabilities.

## References

[B1] Abeti R. Jasoliya M. Al-Mahdawi S. Pook M. Gonzalez-Robles C. Hui C. K. (2022). A drug combination rescues frataxin-dependent neural and cardiac pathophysiology in FA models. Front. Mol. Biosci. 9, 830650. 10.3389/fmolb.2022.830650 35664670 PMC9160322

[B2] Au W. H. Miller-Fleming L. Sanchez-Martinez A. Lee J. A. Twyning M. J. Prag H. A. (2024). Activation of the Keap1/Nrf2 pathway suppresses mitochondrial dysfunction, oxidative stress, and motor phenotypes in C9orf72 ALS/FTD models. Life Sci. Alliance 7 (9), e202402853. 10.26508/lsa.202402853 38906677 PMC11192839

[B3] Babaei H. Kheirollah A. Ranjbaran M. Cheraghzadeh M. Sarkaki A. Adelipour M. (2023). Preconditioning adipose-derived mesenchymal stem cells with dimethyl fumarate promotes their therapeutic efficacy in the brain tissues of rats with Alzheimer's disease. Biochem. Biophys. Res. Commun. 672, 120–127. 10.1016/j.bbrc.2023.06.045 37348174

[B4] Ban T. Kohno H. Ishihara T. Ishihara N. (2018). Relationship between OPA1 and cardiolipin in mitochondrial inner-membrane fusion. Biochim. Biophys. Acta Bioenerg. 1859 (9), 951–957. 10.1016/j.bbabio.2018.05.016 29852142

[B5] Berman S. B. Hastings T. G. (1999). Dopamine oxidation alters mitochondrial respiration and induces permeability transition in brain mitochondria: implications for Parkinson's disease. J. Neurochem. 73 (3), 1127–1137. 10.1046/j.1471-4159.1999.0731127.x 10461904

[B6] Birsa N. Norkett R. Wauer T. Mevissen T. E. Wu H. C. Foltynie T. (2014). Lysine 27 ubiquitination of the mitochondrial transport protein Miro is dependent on serine 65 of the Parkin ubiquitin ligase. J. Biol. Chem. 289 (21), 14569–14582. 10.1074/jbc.M114.563031 24671417 PMC4031514

[B7] Blair H. A. (2018). Dimethyl fumarate: a review in moderate to severe plaque psoriasis. Drugs 78 (1), 123–130. 10.1007/s40265-017-0854-6 29236231

[B8] Bock F. J. Tait S. W. G. (2020). Mitochondria as multifaceted regulators of cell death. Nat. Rev. Mol. Cell Biol. 21, 85–100. 10.1038/s41580-019-0173-8 31636403

[B9] Boczkowski J. Lisdero C. L. Lanone S. Samb A. Carreras M. C. Boveris A. (1999). Endogenous peroxynitrite mediates mitochondrial dysfunction in rat diaphragm during endotoxemia. FASEB J. 13 (12), 1637–1646. 10.1096/fasebj.13.12.1637 10463956

[B10] Bouchez C. Devin A. (2019). Mitochondrial biogenesis and mitochondrial reactive oxygen species (ROS): a complex relationship regulated by the cAMP/PKA signaling pathway. Cells 8 (4), 287. 10.3390/cells8040287 30934711 PMC6523352

[B11] Bresciani G. Manai F. Davinelli S. Tucci P. Saso L. Amadio M. (2023). Novel potential pharmacological applications of dimethyl fumarate-an overview and update. Front. Pharmacol. 14, 1264842. 10.3389/fphar.2023.1264842 37745068 PMC10512734

[B12] Brown G. C. Borutaite V. (2007). Nitric oxide and mitochondrial respiration in the heart. Cardiovasc Res. 75 (2), 283–290. 10.1016/j.cardiores.2007.03.022 17466959

[B13] Burness C. B. Deeks E. D. (2014). Dimethyl fumarate: a review of its use in patients with relapsing-remitting multiple sclerosis. CNS Drugs 28 (4), 373–387. 10.1007/s40263-014-0155-5 24623127

[B14] Campbell C. J. Pall A. E. Naik A. R. Thompson L. N. Stemmler T. L. (2021). Molecular details of the frataxin-scaffold interaction during mitochondrial Fe-S cluster assembly. Int. J. Mol. Sci. 22 (11), 6006. 10.3390/ijms22116006 34199378 PMC8199681

[B15] Campbell N. K. Fitzgerald H. K. Dunne A. (2021). Regulation of inflammation by the antioxidant haem oxygenase 1. Nat. Rev. Immunol. 21 (7), 411–425. 10.1038/s41577-020-00491-x 33514947

[B16] Cantó C. Gerhart-Hines Z. Feige J. N. Lagouge M. Noriega L. Milne J. C. (2009). AMPK regulates energy expenditure by modulating NAD+ metabolism and SIRT1 activity. Nature 458 (7241), 1056–1060. 10.1038/nature07813 19262508 PMC3616311

[B17] Cao Z. Lindsay J. G. Isaacs N. W. (2007). Mitochondrial peroxiredoxins. Subcell. Biochem. 44, 295–315. 10.1007/978-1-4020-6051-9_14 18084900

[B147] Cardozo G. Mastrogiovanni M. Zeida A. Viera N. Radi R. Reyes A. M. (2023). Mitochondrial peroxiredoxin 3 is rapidly oxidized and hyperoxidized by fatty acid hydroperoxides. Antioxidants (Basel) 12 (2), 408. 10.3390/antiox12020408 36829967 PMC9952270

[B18] Carvalho A. N. Firuzi O. Gama M. J. Horssen J. V. Saso L. (2017). Oxidative stress and antioxidants in neurological diseases: is there still hope? Curr. Drug Targets 18 (6), 705–718. 10.2174/1389450117666160401120514 27033198

[B19] Chen H. Assmann J. C. Krenz A. Rahman M. Grimm M. Karsten C. M. (2014). Hydroxycarboxylic acid receptor 2 mediates dimethyl fumarate's protective effect in EAE. J. Clin. Invest. 124 (5), 2188–2192. 10.1172/JCI72151 24691444 PMC4001545

[B20] Chen W. Zhao H. Li Y. (2023). Mitochondrial dynamics in health and disease: mechanisms and potential targets. Signal Transduct. Target Ther. 8 (1), 333. 10.1038/s41392-023-01547-9 37669960 PMC10480456

[B21] Chen T. H. Wang H. C. Chang C. J. Lee S. Y. (2024). Mitochondrial glutathione in cellular redox homeostasis and disease manifestation. Int. J. Mol. Sci. 25 (2), 1314. 10.3390/ijms25021314 38279310 PMC10816320

[B22] Choi H. J. Lee S. Y. Cho Y. No H. Kim S. W. Hwang O. (2006). Tetrahydrobiopterin causes mitochondrial dysfunction in dopaminergic cells: implications for Parkinson's disease. Neurochem. Int. 48 (4), 255–262. 10.1016/j.neuint.2005.10.011 16343695

[B23] Cobley J. N. Fiorello M. L. Bailey D. M. (2018). 13 reasons why the brain is susceptible to oxidative stress. Redox Biol. 15, 490–503. 10.1016/j.redox.2018.01.008 29413961 PMC5881419

[B24] Cordaro M. Casili G. Paterniti I. Cuzzocrea S. Esposito E. (2017). Fumaric acid esters attenuate secondary degeneration after spinal cord injury. J. Neurotrauma 34 (21), 3027–3040. 10.1089/neu.2016.4678 27889959

[B25] Czabotar P. E. Garcia-Saez A. J. (2023). Mechanisms of BCL-2 family proteins in mitochondrial apoptosis. Nat. Rev. Mol. Cell Biol. 24 (10), 732–748. 10.1038/s41580-023-00629-4 37438560

[B26] De Armas M. I. Esteves R. Viera N. Reyes A. M. Mastrogiovanni M. Alegria T. G. P. (2019). Rapid peroxynitrite reduction by human peroxiredoxin 3: implications for the fate of oxidants in mitochondria. Free Radic. Biol. Med. 130, 369–378. 10.1016/j.freeradbiomed.2018.10.451 30391677

[B27] de Brito O. M. Scorrano L. (2010). An intimate liaison: spatial organization of the endoplasmic reticulum-mitochondria relationship. EMBO J. 29 (16), 2715–2723. 10.1038/emboj.2010.177 20717141 PMC2924651

[B28] De Nuccio C. Bernardo A. Troiano C. Brignone M. S. Falchi M. Greco A. (2020). NRF2 and PPAR-γ pathways in oligodendrocyte progenitors: focus on ROS protection, mitochondrial biogenesis and promotion of cell differentiation. Int. J. Mol. Sci. 21 (19), 7216. 10.3390/ijms21197216 33003644 PMC7583077

[B29] Dho S. H. Cho M. Woo W. Jeong S. Kim L. K. (2025). Caspases as master regulators of programmed cell death: apoptosis, pyroptosis and beyond. Exp. Mol. Med. 57, 1121–1132. 10.1038/s12276-025-01470-9 40555741 PMC12229594

[B30] Dinoto A. Sartori A. Cheli M. Pasquin F. Baldini S. Bratina A. (2022). Lymphopenia during treatment with dimethyl fumarate in patients with multiple sclerosis: prevalence, predicting factors and clinical outcomes. Mult. Scler. Relat. Disord. 57, 103357. 10.1016/j.msard.2021.103357 35158466

[B31] Dorn G. W. (2020). Mitofusins as mitochondrial anchors and tethers. J. Mol. Cell Cardiol. 142, 146–153. 10.1016/j.yjmcc.2020.04.016 32304672 PMC7275906

[B32] Farge G. Mehmedovic M. Baclayon M. van den Wildenberg S. M. Roos W. H. Gustafsson C. M. (2014). In vitro-reconstituted nucleoids can block mitochondrial DNA replication and transcription. Cell Rep. 8 (1), 66–74. 10.1016/j.celrep.2014.05.046 24981867

[B33] Fernandez-Marcos P. J. Auwerx J. (2011). Regulation of PGC-1α, a nodal regulator of mitochondrial biogenesis. Am. J. Clin. Nutr. 93 (4), 884S–890S. 10.3945/ajcn.110.001917 21289221 PMC3057551

[B34] Ferrer-Sueta G. Campolo N. Trujillo M. Bartesaghi S. Carballal S. Romero N. (2018). Biochemistry of peroxynitrite and protein tyrosine nitration. Chem. Rev. 118 (3), 1338–1408. 10.1021/acs.chemrev.7b00568 29400454

[B35] Forman H. J. Zhang H. (2021). Targeting oxidative stress in disease: promise and limitations of antioxidant therapy. Nat. Rev. Drug Discov. 20 (9), 689–709. 10.1038/s41573-021-00233-1 34194012 PMC8243062

[B36] Fox R. J. Kita M. Cohan S. L. Henson L. J. Zambrano J. Scannevin R. H. (2014). BG-12 (dimethyl fumarate): a review of mechanism of action, efficacy, and safety. Curr. Med. Res. Opin. 30 (2), 251–262. 10.1185/03007995.2013.849236 24131282

[B37] Friedman J. R. Lackner L. L. West M. DiBenedetto J. R. Nunnari J. Voeltz G. K. (2011). ER tubules mark sites of mitochondrial division. Science 334 (6054), 358–362. 10.1126/science.1207385 21885730 PMC3366560

[B38] Fulco M. Cen Y. Zhao P. Hoffman E. P. McBurney M. W. Sauve A. A. (2008). Glucose restriction inhibits skeletal myoblast differentiation by activating SIRT1 through AMPK-mediated regulation of Nampt. Dev. Cell 14 (5), 661–673. 10.1016/j.devcel.2008.02.004 18477450 PMC2431467

[B39] Gan Z. Y. Callegari S. Cobbold S. A. Cotton T. R. Mlodzianoski M. J. Schubert A. F. (2022). Activation mechanism of PINK1. Nature 602 (7896), 328–335. 10.1038/s41586-021-04340-2 34933320 PMC8828467

[B40] Gao S. J. Li D. Y. Liu D. Q. Sun J. Zhang L. Q. Wu J. Y. (2022). Dimethyl fumarate attenuates pain behaviors in osteoarthritis rats *via* induction of Nrf2-Mediated mitochondrial biogenesis. Mol. Pain 18, 17448069221124920. 10.1177/17448069221124920 36065971 PMC9478692

[B41] Gao M. Z. Zeng J. Y. Chen X. J. Shi L. Hong F. Y. Lin M. (2024). Dimethyl fumarate ameliorates oxidative stress-induced acute kidney injury after traumatic brain injury by activating Keap1-Nrf2/HO-1 signaling pathway. Heliyon 10 (11), e32377. 10.1016/j.heliyon.2024.e32377 38947486 PMC11214498

[B42] Gerhart-Hines Z. Rodgers J. T. Bare O. Lerin C. Kim S. H. Mostoslavsky R. (2007). Metabolic control of muscle mitochondrial function and fatty acid oxidation through SIRT1/PGC-1alpha. EMBO J. 26 (7), 1913–1923. 10.1038/sj.emboj.7601633 17347648 PMC1847661

[B43] Gillard G. O. Collette B. Anderson J. Chao J. Scannevin R. H. Huss D. J. (2015). DMF, but not other fumarates, inhibits NF-κB activity *in vitro* in an Nrf2-independent manner. J. Neuroimmunol. 283, 74–85. 10.1016/j.jneuroim.2015.04.006 26004161

[B44] Glover H. L. Schreiner A. Dewson G. Tait S. W. G. (2024). Mitochondria and cell death. Nat. Cell Biol. 26 (9), 1434–1446. 10.1038/s41556-024-01429-4 38902422

[B45] Gold R. Kappos L. Arnold D. L. Bar-Or A. Giovannoni G. Selmaj K. (2012). Placebo-controlled phase 3 study of oral BG-12 for relapsing multiple sclerosis. N. Engl. J. Med. 367 (12), 1098–1107. 10.1056/NEJMoa1114287 22992073

[B46] Hall-Younger E. Tait S. W. (2025). Mitochondria and cell death signalling. Curr. Opin. Cell Biol. 94, 102510. 10.1016/j.ceb.2025.102510 40215948

[B47] Halliwell B. (2006). Oxidative stress and neurodegeneration: where are we now? J. Neurochem. 97 (6), 1634–1658. 10.1111/j.1471-4159.2006.03907.x 16805774

[B48] Harper J. W. Ordureau A. Heo J. M. (2018). Building and decoding ubiquitin chains for mitophagy. Nat. Rev. Mol. Cell Biol. 19 (2), 93–108. 10.1038/nrm.2017.129 29358684

[B49] Harrington J. S. Ryter S. W. Plataki M. Price D. R. Choi A. M. K. (2023). Mitochondria in health, disease, and aging. Physiol. Rev. 103 (4), 2349–2422. 10.1152/physrev.00058.2021 37021870 PMC10393386

[B50] Hayashi G. Jasoliya M. Sahdeo S. Saccà F. Pane C. Filla A. (2017). Dimethyl fumarate mediates Nrf2-dependent mitochondrial biogenesis in mice and humans. Hum. Mol. Genet. 26 (15), 2864–2873. 10.1093/hmg/ddx167 28460056 PMC6251607

[B51] Holley A. K. Bakthavatchalu V. Velez-Roman J. M. St Clair D. K. (2011). Manganese superoxide dismutase: guardian of the powerhouse. Int. J. Mol. Sci. 12 (10), 7114–7162. 10.3390/ijms12107114 22072939 PMC3211030

[B52] Huang M. L. Chiang S. Kalinowski D. S. Bae D. H. Sahni S. Richardson D. R. (2019). The role of the antioxidant response in mitochondrial dysfunction in degenerative diseases: cross-talk between antioxidant defense, autophagy, and apoptosis. Oxid. Med. Cell Longev. 2019, 6392763. 10.1155/2019/6392763 31057691 PMC6476015

[B53] Hui C. K. Dedkova E. N. Montgomery C. Cortopassi G. (2021). Dimethyl fumarate dose-dependently increases mitochondrial gene expression and function in muscle and brain of Friedreich’s ataxia model mice. Hum. Mol. Genet. 29 (24), 3954–3965. 10.1093/hmg/ddaa282 33432356 PMC8485216

[B54] Hull T. D. Boddu R. Guo L. Tisher C. C. Traylor A. M. Patel B. (2016). Heme oxygenase-1 regulates mitochondrial quality control in the heart. JCI Insight 1 (2), e85817. 10.1172/jci.insight.85817 27110594 PMC4838906

[B55] Jäger S. Handschin C. St-Pierre J. Spiegelman B. M. (2007). AMP-activated protein kinase (AMPK) action in skeletal muscle *via* direct phosphorylation of PGC-1alpha. Proc. Natl. Acad. Sci. U. S. A. 104 (29), 12017–12022. 10.1073/pnas.0705070104 17609368 PMC1924552

[B56] Jana S. Sinha M. Chanda D. Roy T. Banerjee K. Munshi S. (2011). Mitochondrial dysfunction mediated by quinone oxidation products of dopamine: implications in dopamine cytotoxicity and pathogenesis of Parkinson’s disease. Biochim. Biophys. Acta 1812 (6), 663–673. 10.1016/j.bbadis.2011.02.013 21377526

[B57] Jasoliya M. Sacca F. Sahdeo S. Chedin F. Pane C. Brescia Morra V. (2019). Dimethyl fumarate dosing in humans increases frataxin expression: a potential therapy for Friedreich’s ataxia. PLoS One 14 (6), e0217776. 10.1371/journal.pone.0217776 31158268 PMC6546270

[B58] Ji W. K. Hatch A. L. Merrill R. A. Strack S. Higgs H. N. (2015). Actin filaments target the oligomeric maturation of the dynamin GTPase Drp1 to mitochondrial fission sites. Elife 4, e11553. 10.7554/eLife.11553 26609810 PMC4755738

[B59] Johansen T. Lamark T. (2020). Selective autophagy: ATG8 family proteins, LIR Motifs and Cargo receptors. J. Mol. Biol. 432 (1), 80–103. 10.1016/j.jmb.2019.07.016 31310766

[B60] Jornayvaz F. R. Shulman G. I. (2010). Regulation of mitochondrial biogenesis. Essays Biochem. 47, 69–84. 10.1042/bse0470069 20533901 PMC3883043

[B61] Joubert F. Puff N. (2021). Mitochondrial cristae Architecture and functions: lessons from minimal model systems. Membr. (Basel) 11 (7), 465. 10.3390/membranes11070465 34201754 PMC8306996

[B62] Khot M. Sood A. Tryphena K. P. Pinjala P. Srivastava S. Singh S. B. (2023). Dimethyl fumarate ameliorates parkinsonian pathology by modulating autophagy and apoptosis *via* Nrf2-TIGAR-LAMP2/Cathepsin D axis. Brain Res. 1815, 148462. 10.1016/j.brainres.2023.148462 37315723

[B63] Kondadi A. K. Anand R. Reichert A. S. (2020). Cristae membrane dynamics - a paradigm change. Trends Cell Biol. 30 (12), 923–936. 10.1016/j.tcb.2020.08.008 32978040

[B64] Kornberg M. D. Bhargava P. Kim P. M. Putluri V. Snowman A. M. Putluri N. (2018). Dimethyl fumarate targets GAPDH and aerobic glycolysis to modulate immunity. Science 360 (6387), 449–453. 10.1126/science.aan4665 29599194 PMC5924419

[B65] Kraus F. Roy K. Pucadyil T. J. Ryan M. T. (2021). Function and regulation of the divisome for mitochondrial fission. Nature 590, 57–66. 10.1038/s41586-021-03214-x 33536648

[B66] Kukat C. Davies K. M. Wurm C. A. Spåhr H. Bonekamp N. A. Kühl I. (2015). Cross-strand binding of TFAM to a single mtDNA molecule forms the mitochondrial nucleoid. Proc. Natl. Acad. Sci. U. S. A. 112 (36), 11288–11293. 10.1073/pnas.1512131112 26305956 PMC4568684

[B67] Kukulage D. S. K. Matarage Don N. N. J. Ahn Y. H. (2022). Emerging chemistry and biology in protein glutathionylation. Curr. Opin. Chem. Biol. 71, 102221. 10.1016/j.cbpa.2022.102221 36223700 PMC9844265

[B68] Lacza Z. Pankotai E. Busija D. W. (2009). Mitochondrial nitric oxide synthase: current concepts and controversies. Front. Biosci. (Landmark Ed.) 14 (12), 4436–4443. 10.2741/3539 19273361 PMC4570492

[B69] Lambert A. J. Brand M. D. (2009). Reactive oxygen species production by mitochondria. Methods Mol. Biol. 554, 165–181. 10.1007/978-1-59745-521-3_11 19513674

[B70] Lategan T. W. Wang L. Sprague T. N. Rousseau F. S. (2021). Pharmacokinetics and bioavailability of monomethyl fumarate following a single oral dose of bafiertam™ (Monomethyl fumarate) or tecfidera® (Dimethyl fumarate). CNS Drugs 35 (5), 567–574. 10.1007/s40263-021-00799-9 33797063 PMC8144082

[B71] Lee Y. M. He W. Liou Y. C. (2021). The redox language in neurodegenerative diseases: oxidative post-translational modifications by hydrogen peroxide. Cell Death Dis. 12 (1), 58. 10.1038/s41419-020-03355-3 33431811 PMC7801447

[B72] Li Y. Rasheed M. Liu J. Chen Z. Deng Y. (2024). Deciphering the molecular nexus: an in-depth review of mitochondrial pathways and their role in cell death crosstalk. Cells 13 (10), 863. 10.3390/cells13100863 38786088 PMC11119937

[B73] Li Z. Z. Xiao H. X. Hu J. J. Xie W. Wang Z. X. Pan Y. P. (2025). The mechanisms and implications of cardiolipin in the regulation of cell death. Cell Biochem. Funct. 43 (3), e70066. 10.1002/cbf.70066 40103184

[B74] Liang G. Chai J. Ng H. S. Tremlett H. (2020). Safety of dimethyl fumarate for multiple sclerosis: a systematic review and meta-analysis. Mult. Scler. Relat. Disord. 46, 102566. 10.1016/j.msard.2020.102566 33296968

[B75] Liebmann M. Korn L. Janoschka C. Albrecht S. Lauks S. Herrmann A. M. (2021). Dimethyl fumarate treatment restrains the antioxidative capacity of T cells to control autoimmunity. Brain 144 (10), 3126–3141. 10.1093/brain/awab307 34849598 PMC8634070

[B76] Lin M. M. Liu N. Qin Z. H. Wang Y. (2022). Mitochondrial-derived damage-associated molecular patterns amplify neuroinflammation in neurodegenerative diseases. Acta Pharmacol. Sin. 43 (10), 2439–2447. 10.1038/s41401-022-00879-6 35233090 PMC9525705

[B77] Lin H. Wang L. Jiang X. Wang J. (2024). Glutathione dynamics in subcellular compartments and implications for drug development. Curr. Opin. Chem. Biol. 81, 102505. 10.1016/j.cbpa.2024.102505 39053236 PMC11722958

[B78] Liu D. Liu Z. Hu Y. Xiong W. Wang D. Zeng Z. (2025). MOMP: a critical event in cell death regulation and anticancer treatment. Biochim. Biophys. Acta Rev. Cancer 1880, 189280. 10.1016/j.bbcan.2025.189280 39947442

[B79] Lonkar N. Latz E. McManus R. M. (2025). Neuroinflammation and immunometabolism in neurodegenerative diseases. Curr. Opin. Neurol. 38 (2), 163–171. 10.1097/WCO.0000000000001356 39936491

[B80] Luchkova A. Mata A. Cadenas S. (2024). Nrf2 as a regulator of energy metabolism and mitochondrial function. FEBS Lett. 598 (17), 2092–2105. 10.1002/1873-3468.14993 39118293

[B81] Magalhães R. Bourgin J. Boumezbeur F. Marques P. Bottlaender M. Poupon C. (2017). White matter changes in microstructure associated with a maladaptive response to stress in rats. Transl. Psychiatry 7 (1), e1009. 10.1038/tp.2016.283 28117841 PMC5545740

[B82] Mailloux R. J. (2018). Mitochondrial antioxidants and the maintenance of cellular hydrogen peroxide levels. Oxid. Med. Cell Longev. 2018, 7857251. 10.1155/2018/7857251 30057684 PMC6051038

[B83] Majkutewicz I. (2022). Dimethyl fumarate: a review of preclinical efficacy in models of neurodegenerative diseases. Eur. J. Pharmacol. 926, 175025. 10.1016/j.ejphar.2022.175025 35569547

[B84] Manai F. Zanoletti L. Arfini D. Micco S. G. Gjyzeli A. Comincini S. (2023). Dimethyl fumarate and intestine: from main suspect to potential ally against gut disorders. Int. J. Mol. Sci. 24 (12), 9912. 10.3390/ijms24129912 37373057 PMC10298566

[B85] Manor U. Bartholomew S. Golani G. Christenson E. Kozlov M. Higgs H. (2015). A mitochondria-anchored isoform of the actin-nucleating spire protein regulates mitochondrial division. Elife 4, e08828. 10.7554/eLife.08828 26305500 PMC4574297

[B86] Mansilla M. J. Navarro-Barriuso J. Presas-Rodríguez S. Teniente-Serra A. Quirant-Sánchez B. Ramo-Tello C. (2019). Optimal response to dimethyl fumarate is mediated by a reduction of Th1-like Th17 cells after 3 months of treatment. CNS Neurosci. Ther. 25 (9), 995–1005. 10.1111/cns.13142 31066225 PMC6698982

[B87] Marí M. de Gregorio E. de Dios C. Roca-Agujetas V. Cucarull B. Tutusaus A. (2020). Mitochondrial glutathione: recent insights and role in disease. Antioxidants (Basel) 9 (10), 909. 10.3390/antiox9100909 32987701 PMC7598719

[B88] Martinez A. N. Tortelote G. G. Pascale C. L. Ekanem U. I. Leite A. P. O. McCormack I. G. (2024). Dimethyl fumarate mediates sustained vascular smooth muscle cell remodeling in a mouse model of cerebral aneurysm. Antioxidants (Basel) 13 (7), 773. 10.3390/antiox13070773 39061841 PMC11274241

[B89] Masuda D. Nakanishi I. Ohkubo K. Ito H. Matsumoto K. I. Ichikawa H. (2024). Mitochondria play essential roles in intracellular protection against oxidative stress-which molecules among the ROS generated in the mitochondria can escape the mitochondria and contribute to signal activation in cytosol? Biomolecules 14 (1), 128. 10.3390/biom14010128 38275757 PMC10813015

[B90] Matsumoto G. Wada K. Okuno M. Kurosawa M. Nukina N. (2011). Serine 403 phosphorylation of p62/SQSTM1 regulates selective autophagic clearance of ubiquitinated proteins. Mol. Cell 44 (2), 279–289. 10.1016/j.molcel.2011.07.039 22017874

[B91] Meiser J. Weindl D. Hiller K. (2013). Complexity of dopamine metabolism. Cell Commun. Signal 11 (1), 34. 10.1186/1478-811X-11-34 23683503 PMC3693914

[B92] Murphy M. P. (2009). How mitochondria produce reactive oxygen species. Biochem. J. 417 (1), 1–13. 10.1042/BJ20081386 19061483 PMC2605959

[B93] Narine M. Colognato H. (2022). Current insights into oligodendrocyte metabolism and its power to sculpt the Myelin landscape. Front. Cell Neurosci. 16, 892968. 10.3389/fncel.2022.892968 35573837 PMC9097137

[B94] Navarro E. Gonzalez-Lafuente L. Pérez-Liébana I. Buendia I. López-Bernardo E. Sánchez-Ramos C. (2017). Heme-oxygenase I and PCG-1α regulate mitochondrial biogenesis *via* microglial activation of Alpha7 nicotinic acetylcholine receptors using PNU282987. Antioxid. Redox Signal 27 (2), 93–105. 10.1089/ars.2016.6698 27554853

[B95] Papa S. Martino P. L. Capitanio G. Gaballo A. De Rasmo D. Signorile A. (2012). The oxidative phosphorylation system in mammalian mitochondria. Adv. Exp. Med. Biol. 942, 3–37. 10.1007/978-94-007-2869-1_1 22399416

[B96] Paradies G. Paradies V. Ruggiero F. M. Petrosillo G. (2019). Role of cardiolipin in mitochondrial function and dynamics in health and disease: molecular and pharmacological aspects. Cells 8 (7), 728. 10.3390/cells8070728 31315173 PMC6678812

[B97] Peggion C. Calì T. Brini M. (2024). Mitochondria dysfunction and neuroinflammation in neurodegeneration: who comes first? Antioxidants (Basel) 13 (2), 240. 10.3390/antiox13020240 38397838 PMC10885966

[B98] Petrillo S. D’Amico J. La Rosa P. Bertini E. S. Piemonte F. (2019). Targeting NRF2 for the treatment of Friedreich’s ataxia: a comparison among drugs. Int. J. Mol. Sci. 20 (20), 5211. 10.3390/ijms20205211 31640150 PMC6829337

[B99] Pfanner N. Warscheid B. Wiedemann N. (2019). Mitochondrial proteins: from biogenesis to functional networks. Nat. Rev. Mol. Cell Biol. 20 (5), 267–284. 10.1038/s41580-018-0092-0 30626975 PMC6684368

[B100] Piel S. McManus M. J. Heye K. N. Beaulieu F. Fazelinia H. Janowska J. I. (2024). Effect of dimethyl fumarate on mitochondrial metabolism in a pediatric porcine model of asphyxia-induced in-hospital cardiac arrest. Sci. Rep. 14 (1), 13852. 10.1038/s41598-024-64317-9 38879681 PMC11180202

[B101] Pinjala P. Tryphena K. P. Kulkarni A. Goswami P. G. Khatri D. K. (2024). Dimethyl fumarate exerts a neuroprotective effect by enhancing mitophagy *via* the NRF2/BNIP3/PINK1 axis in the MPP+ iodide-induced Parkinson's Disease mice model. J. Alzheimers Dis. Rep. 8 (1), 329–344. 10.3233/ADR-230128 38405353 PMC10894611

[B102] Rajput M. S. Nirmal N. P. Rathore D. Dahima R. (2020). Dimethyl fumarate mitigates tauopathy in Aβ-Induced neuroblastoma SH-SY5Y cells. Neurochem. Res. 45 (11), 2641–2652. 10.1007/s11064-020-03115-x 32816241

[B103] Ranea-Robles P. Launay N. Ruiz M. Calingasan N. Y. Dumont M. Naudí A. (2018). Aberrant regulation of the GSK-3β/NRF2 axis unveils a novel therapy for adrenoleukodystrophy. EMBO Mol. Med. 10 (8), e8604. 10.15252/emmm.201708604 29997171 PMC6079538

[B104] Rasheed MRHA Tarjan G. (2018). Succinate dehydrogenase complex: an updated review. Arch. Pathol. Lab. Med. 142 (12), 1564–1570. 10.5858/arpa.2017-0285-RS 30289269

[B105] Rodgers J. T. Lerin C. Haas W. Gygi S. P. Spiegelman B. M. Puigserver P. (2005). Nutrient control of glucose homeostasis through a complex of PGC-1alpha and SIRT1. Nature 434 (7029), 113–118. 10.1038/nature03354 15744310

[B106] Rosito M. Testi C. Parisi G. Cortese B. Baiocco P. Di Angelantonio S. (2020). Exploring the use of Dimethyl fumarate as microglia modulator for neurodegenerative diseases treatment. Antioxidants (Basel) 9 (8), 700. 10.3390/antiox9080700 32756501 PMC7465338

[B107] Ross D. Siegel D. (2021). The diverse functionality of NQO1 and its roles in redox control. Redox Biol. 41, 101950. 10.1016/j.redox.2021.101950 33774477 PMC8027776

[B108] Sadovnikova I. S. Gureev A. P. Ignatyeva D. A. Gryaznova M. V. Chernyshova E. V. Krutskikh E. P. (2021). Nrf2/ARE activators improve memory in aged mice *via* maintaining of mitochondrial quality control of brain and the modulation of gut microbiome. Pharm. (Basel). 14 (7), 607. 10.3390/ph14070607 34201885 PMC8308546

[B109] Sangineto M. Ciarnelli M. Moola A. Naik Bukke V. Cassano T. Villani R. (2024). Krebs cycle derivatives, dimethyl fumarate and itaconate, control metabolic reprogramming in inflammatory human microglia cell line. Mitochondrion 79, 79101966. 10.1016/j.mito.2024.101966 39276907

[B110] Sena L. A. Chandel N. S. (2012). Physiological roles of mitochondrial reactive oxygen species. Mol. Cell 48 (2), 158–167. 10.1016/j.molcel.2012.09.025 23102266 PMC3484374

[B111] Șerban M. Toader C. Covache-Busuioc R. A. (2025). The redox revolution in brain medicine: targeting oxidative stress with AI, multi-omics and mitochondrial therapies for the precision eradication of neurodegeneration. Int. J. Mol. Sci. 26 (15), 7498. 10.3390/ijms26157498 40806624 PMC12347410

[B112] Sghaier R. Nury T. Leoni V. Caccia C. Pais De Barros J. P. Cherif A. (2019). Dimethyl fumarate and monomethyl fumarate attenuate oxidative stress and mitochondrial alterations leading to oxiapoptophagy in 158N murine oligodendrocytes treated with 7β-hydroxycholesterol. J. Steroid Biochem. Mol. Biol. 194, 105432. 10.1016/j.jsbmb.2019.105432 31344443

[B113] Shavakandi S. M. Ranjbaran M. Nabavizadeh F. Vali R. Sehati F. Ashabi G. (2022). Dimethyl fumarate protects the aged brain following chronic cerebral hypoperfusion-related ischemia in rats in Nrf2-dependent manner. Nutr. Neurosci. 25 (10), 2100–2110. 10.1080/1028415X.2021.1940429 34148507

[B114] Shen Y. Jiang W. L. Li X. Cao A. L. Li D. Li S. Z. (2023). Mitochondrial dynamics in neurological diseases: a narrative review. Ann. Transl. Med. 11 (6), 264. 10.21037/atm-22-2401 37082676 PMC10113088

[B115] Shergalis A. G. Hu S. Bankhead A. Neamati N. (2020). Role of the ERO1-PDI interaction in oxidative protein folding and disease. Pharmacol. Ther. 210, 107525. 10.1016/j.pharmthera.2020.107525 32201313 PMC7316501

[B116] Sies H. (2017). Hydrogen peroxide as a central redox signaling molecule in physiological oxidative stress: oxidative eustress. Redox Biol. 11, 613–619. 10.1016/j.redox.2016.12.035 28110218 PMC5256672

[B117] Sies H. Jones D. P. (2020). Reactive oxygen species (ROS) as pleiotropic physiological signalling agents. Nat. Rev. Mol. Cell Biol. 21 (7), 363–383. 10.1038/s41580-020-0230-3 32231263

[B118] Sies H. Berndt C. Jones D. P. (2017). Oxidative stress. Annu. Rev. Biochem. 86, 715–748. 10.1146/annurev-biochem-061516-045037 28441057

[B119] Sies H. Belousov V. V. Chandel N. S. Davies M. J. Jones D. P. Mann G. E. (2022). Defining roles of specific reactive oxygen species (ROS) in cell biology and physiology. Nat. Rev. Mol. Cell Biol. 23 (7), 499–515. 10.1038/s41580-022-00456-z 35190722

[B120] Sies H. Mailloux R. J. Jakob U. (2024). Fundamentals of redox regulation in biology. Nat. Rev. Mol. Cell Biol. 25 (9), 701–719. 10.1038/s41580-024-00730-2 38689066 PMC11921270

[B121] Silva A. Pereira M. Carrascal M. A. Brites G. Neves B. Moreira P. (2020). Calcium modulation, anti-oxidant and anti-inflammatory effect of skin allergens targeting the Nrf2 signaling pathway in Alzheimer’s disease cellular models. Int. J. Mol. Sci. 21 (20), 7791. 10.3390/ijms21207791 33096789 PMC7594024

[B122] Singh N. Saha L. Kumari P. Singh J. Bhatia A. Banerjee D. (2019). Effect of dimethyl fumarate on neuroinflammation and apoptosis in pentylenetetrazol kindling model in rats. Brain Res. Bull. 144, 233–245. 10.1016/j.brainresbull.2018.11.013 30472152

[B123] Smith F. M. Kosman D. J. (2020). Molecular defects in Friedreich’s ataxia: convergence of oxidative stress and cytoskeletal abnormalities. Front. Mol. Biosci. 7, 569293. 10.3389/fmolb.2020.569293 33263002 PMC7686857

[B124] Solleiro-Villavicencio H. Rivas-Arancibia S. (2018). Effect of chronic oxidative stress on neuroinflammatory response mediated by CD4+T cells in neurodegenerative diseases. Front. Cell Neurosci. 12, 114. 10.3389/fncel.2018.00114 29755324 PMC5934485

[B125] Song N. Mei S. Wang X. Hu G. Lu M. (2024). Focusing on mitochondria in the brain: from biology to therapeutics. Transl. Neurodegener. 13 (1), 23. 10.1186/s40035-024-00409-w 38632601 PMC11022390

[B126] Sun F. Fang M. Zhang H. Song Q. Li S. Li Y. (2024). Drp1: focus on diseases triggered by the mitochondrial pathway. Cell Biochem. Biophys. 82 (2), 435–455. 10.1007/s12013-024-01245-5 38438751

[B127] Szczesny-Malysiak E. Stojak M. Campagna R. Grosicki M. Jamrozik M. Kaczara P. (2020). Bardoxolone methyl displays detrimental effects on endothelial bioenergetics, suppresses endothelial ET-1 release, and increases endothelial permeability in human Microvascular endothelium. Oxid. Med. Cell Longev. 2020, 4678252. 10.1155/2020/4678252 33123312 PMC7584962

[B128] Tanaka A. Cleland M. M. Xu S. Narendra D. P. Suen D. F. Karbowski M. (2010). Proteasome and p97 mediate mitophagy and degradation of mitofusins induced by Parkin. J. Cell Biol. 191 (7), 1367–1380. 10.1083/jcb.201007013 21173115 PMC3010068

[B129] Tang J. X. Thompson K. Taylor R. W. Oláhová M. (2020). Mitochondrial OXPHOS biogenesis: co-regulation of protein synthesis, import, and assembly pathways. Int. J. Mol. Sci. 21 (11), 3820. 10.3390/ijms21113820 32481479 PMC7312649

[B130] Thurston T. L. Ryzhakov G. Bloor S. von Muhlinen N. Randow F. (2009). The TBK1 adaptor and autophagy receptor NDP52 restricts the proliferation of ubiquitin-coated bacteria. Nat. Immunol. 10 (11), 1215–1221. 10.1038/ni.1800 19820708

[B131] Tilokani L. Nagashima S. Paupe V. Prudent J. (2018). Mitochondrial dynamics: overview of molecular mechanisms. Essays Biochem. 62 (3), 341–360. 10.1042/EBC20170104 30030364 PMC6056715

[B132] Trofin D. M. Sardaru D. P. Trofin D. Onu I. Tutu A. Onu A. (2025). Oxidative stress in brain function. Antioxidants (Basel) 14 (3), 297. 10.3390/antiox14030297 40227270 PMC11939459

[B133] Umek N. Geršak B. Vintar N. Šoštarič M. Mavri J. (2018). Dopamine autoxidation is controlled by acidic pH. Front. Mol. Neurosci. 11, 467. 10.3389/fnmol.2018.00467 30618616 PMC6305604

[B134] Üremiş N. Üremiş M. M. (2025). Oxidative/nitrosative stress, apoptosis, and redox signaling: key players in neurodegenerative diseases. J. Biochem. Mol. Toxicol. 39 (1), e70133. 10.1002/jbt.70133 39799559 PMC11725306

[B135] Vašková J. Kočan L. Vaško L. Perjési P. (2023). Glutathione-related enzymes and proteins: a review. Molecules 28 (3), 1447. 10.3390/molecules28031447 36771108 PMC9919958

[B136] Vogler M. Braun Y. Smith V. M. Westhoff M. A. Pereira R. S. Pieper N. M. (2025). The BCL2 family: from apoptosis mechanisms to new advances in targeted therapy. Signal Transduct. Target Ther. 10, 91. 10.1038/s41392-025-02176-0 40113751 PMC11926181

[B137] Wang S. Long H. Hou L. Feng B. Ma Z. Wu Y. (2023). The mitophagy pathway and its implications in human diseases. Signal Transduct. Target Ther. 8 (1), 304. 10.1038/s41392-023-01503-7 37582956 PMC10427715

[B138] Wong Y. C. Holzbaur E. L. (2015). Temporal dynamics of PARK2/Parkin and OPTN/optineurin recruitment during the mitophagy of damaged mitochondria. Autophagy 11 (2), 422–424. 10.1080/15548627.2015.1009792 25801386 PMC4502688

[B139] Wright D. C. Geiger P. C. Han D. H. Jones T. E. Holloszy J. O. (2007). Calcium induces increases in peroxisome proliferator-activated receptor gamma coactivator-1alpha and mitochondrial biogenesis by a pathway leading to p38 mitogen-activated protein kinase activation. J. Biol. Chem. 282 (26), 18793–18799. 10.1074/jbc.M611252200 17488713

[B140] Wu Q. Wang Q. Mao G. Dowling C. A. Lundy S. K. Mao-Draayer Y. (2017). Dimethyl fumarate selectively reduces memory T cells and shifts the balance between Th1/Th17 and Th2 in multiple sclerosis patients. J. Immunol. 198 (8), 3069–3080. 10.4049/jimmunol.1601532 28258191 PMC5464403

[B141] Wu Y. L. Chang J. C. Chao Y. C. Chan H. Hsieh M. Liu C. S. (2022). *In vitro* efficacy and molecular mechanism of curcumin analog in pathological regulation of spinocerebellar ataxia type 3. Antioxidants (Basel) 11 (7), 1389. 10.3390/antiox11071389 35883884 PMC9311745

[B142] Xu L. Peng C. C. Dawson K. Stecher S. Woodworth J. Prakash C. (2023). Metabolism, pharmacokinetics and excretion of [14C]dimethyl fumarate in healthy volunteers: an example of xenobiotic biotransformation following endogenous metabolic pathways. Xenobiotica 53 (3), 163–172. 10.1080/00498254.2023.2217506 37216617

[B143] Zarrouk A. Nury T. Karym E. M. Vejux A. Sghaier R. Gondcaille C. (2017). Attenuation of 7-ketocholesterol-induced overproduction of reactive oxygen species, apoptosis, and autophagy by dimethyl fumarate on 158N murine oligodendrocytes. J. Steroid Biochem. Mol. Biol. 169, 29–38. 10.1016/j.jsbmb.2016.02.024 26921765

[B144] Zhang H. Forman H. J. (2012). Glutathione synthesis and its role in redox signaling. Semin. Cell Dev. Biol. 23 (7), 722–728. 10.1016/j.semcdb.2012.03.017 22504020 PMC3422610

[B145] Zorov D. B. Juhaszova M. Sollott S. J. (2014). Mitochondrial reactive oxygen species (ROS) and ROS-induced ROS release. Physiol. Rev. 94 (3), 909–950. 10.1152/physrev.00026.2013 24987008 PMC4101632

[B146] Zorova L. D. Popkov V. A. Plotnikov E. Y. Silachev D. N. Pevzner I. B. Jankauskas S. S. (2018). Mitochondrial membrane potential. Anal. Biochem. 552, 50–59. 10.1016/j.ab.2017.07.009 28711444 PMC5792320

